# Naoxintong capsule decreases circulating exosomes of miR-382-5p to protect LPS-induced vascular endothelial cell injury by targeting *STC1 in vitro*

**DOI:** 10.3389/fphar.2026.1655883

**Published:** 2026-03-09

**Authors:** Ziyan Xu, Hao Wu, Weiyang Fan, Fenzhao Meng, Shuangfei Hu, Muhan Zou, Yixuan Chen, Weiwei Su, Peibo Li

**Affiliations:** Guangdong Engineering and Technology Research Center for Quality and Efficacy Re-Evaluation of Post-Marketed TCM, Guangdong Provincial Key Laboratory of Plant Stress Biology, State Key Laboratory of Biocontrol, School of Life Sciences, Sun Yat-sen University, Guangzhou, China

**Keywords:** circulating exosomes, miR-382-5p, naoxintong capsule (NXT), protective effect, stanniocalcin-1 (STC1), TLR4/TRIF/NF-κB pathway, vascular endothelial injury

## Abstract

**Background:**

Cardiovascular and cerebrovascular diseases, major health threats in aging populations, involve vascular endothelial injury as a key pathological factor. Circulating exosomes, a type of extracellular vesicles involved in substance transport and signal transduction, serve as regulatory mediators in vascular diseases. Naoxintong Capsule (NXT), a traditional Chinese medicine with 3 decades of clinical application, protects vascular diseases via multifaceted mechanisms, yet its circulating exosome-mediated endothelial protection remains unclear.

**Methods:**

Circulating exosomes were isolated from NXT-treated (NXT-Exo) and saline-treated (Ctl-Exo) SD rats. The effect of NXT-Exo and Ctl-Exo on lipopolysaccharide (LPS) induced human microvascular endothelial cells (HMEC-1) injury was studied using molecular biology experiments. RNA-seq and miRNA-omics analysis were performed to elucidate the mechanisms of NXT-Exo. Western blot and enzyme linked immunosorbent assay (ELISA) were used to validate the therapeutic target of NXT-Exo.

**Results:**

NXT-Exo attenuated LPS-induced HMEC-1 injury by suppressing apoptosis, inflammation, oxidative stress, and endothelial dysfunction, while Ctl-Exo showed no effect. RNA-seq revealed the TLR4/TRIF/NF-κB signaling pathway might play a crucial role in NXT-Exo’s effect. miRNA-omics suggested miR-382-5p as a pivotal mediator, and ELISA confirmed that its downregulation contributes to the protective effect of NXT-Exo. Integrated analysis indicated Stanniocalcin-1 (*STC1*) may be a miR-382-5p target. Western blot results showed that STC1 silencing aggravated the pro-injury effects of miR-382-5p.

**Conclusion:**

Our findings elucidated that NXT protected HMEC-1 from injury and dysfunction by downregulating miR-382-5p in circulating exosomes, potentially via targeting *STC1* and inhibiting the TLR4/TRIF/NF-κB pathway.

## Graphical Abstract

**Figure:**
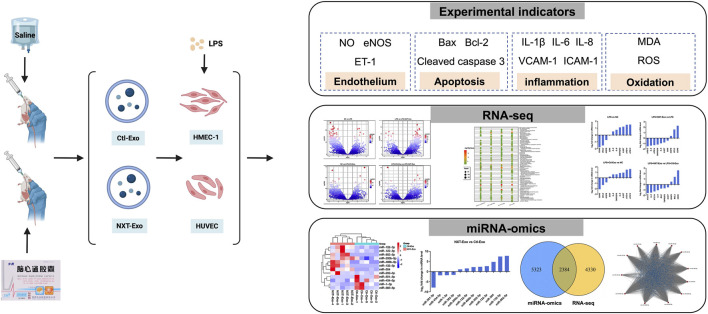

## 1 Introduction

Cardiovascular and cerebrovascular diseases stand as leading causes of mortality globally, with vascular endothelial dysfunction identified as a critical early contributor to their progression. Vascular endothelial cells (VECs) comprise a single-layered, flattened lining along the inner surface of the heart and blood vessels (Wu et al., 2018), orchestrating crucial physiological processes within the organism. Serving as the first biological barrier of molecules and cells in vascular circulation, VECs assume pivotal roles in regulating vascular tone, promoting angiogenesis, maintaining the blood balance between anticoagulation and procoagulation, and constituting a selective permeability barrier (Bloom et al., 2023). The dysfunction of VECs is characterized by vasoconstriction, altered vascular permeability, and dysregulated immune modulation (Evans et al., 2021). Numerous studies have confirmed VECs injury and dysfunction as an early pathological hallmark in conditions such as atherosclerosis, hyperlipidemia, and heart failure (Augustin and Koh, 2024). When the body is infected or tissues are damaged, vascular endothelial injury and dysfunction are the initial factors of cardiovascular and cerebrovascular diseases. Despite significant advancements in drug therapy and surgical interventions in recent years, the prolonged latency period and older age of onset in affected individuals heighten the risks of disease progression and recurrence. Therefore, identifying new therapeutic targets and effective drugs to prevent VECs injury and dysfunction is essential for advancing the prevention and clinical treatment of cardiovascular and cerebrovascular diseases.

Exosomes are extracellular vesicles (EVs) with a diameter of 30∼150 nm, secreted by all types of cells (Zhang et al., 2018). They serve as vehicles for transferring proteins, lipids, mRNA, miRNA, lncRNA, DNA, amino acids and metabolites from donor cells to recipient cells (Han et al., 2022), facilitating intercellular substance exchange and signal transmission (Heo and Kang, 2022). Among these, circulating exosomes represent a subclass capable of entering the blood circulation, directly interacting with VECs, and transporting substances over long distances through the bloodstream to target tissues and organs (Dong et al., 2018). Recent investigations have increasingly unveiled the involvement of circulating exosomes in pathological processes such as cardiovascular diseases (Xiao et al., 2022), neoplastic diseases (Famta et al., 2022) and diabetes (Xue et al., 2022), thus positioning them as potential biomarkers for disease prognosis and prevention (Vaidya and Sugaya, 2020). For instance, studies have shown that the presence of miR-1229-5p in serum EVs could mitigate hypoxic/reoxygenation-induced cardiomyocyte injury (Jin et al., 2022), while long-term exercise-induced circulating exosomes were found to protect the heart from myocardial ischemia/reperfusion injury through exosomal miR-342-5p (Hou et al., 2019). Hu et al. discovered that plasma exosomes treated with emodin could inhibit the NF-κB pathway by regulating PPARγ, thereby reducing alveolar macrophage activation and attenuating pneumonia inflammation (Hu et al., 2022). However, whether circulating exosomes play a role in the repair process of VECs injury and dysfunction remains unclear.

Traditional Chinese medicine (TCM) has garnered increasing attention in clinical disease treatment due to its multifaceted therapeutic targets, minimal toxic side effects, and stable efficacy (Liu et al., 2018). Naoxintong Capsule (NXT) is a commercial Chinese polyherbal preparation (CCPP) widely used in cardiovascular and cerebrovascular disease management in China (Wang et al., 2022). It comprises 16 kinds of traditional Chinese medicine drugs, including *Astragalus mongholicus* Bunge [Fabaceae; astragali radix], *Salvia miltiorrhiza* Bunge [Lamiaceae; salviae miltiorrhizae radix et rhizoma], *Conioselinum anthriscoides* [Apiaceae; chuanxiong rhizoma], *Paeonia lactiflora* Pall. [Paeoniaceae; paeoniae radix rubra], *Prunus persica* (L.) Batsch [Rosaceae; persicae ramulus], *Carthamus tinctorius* L. [Asteraceae; carthami flos], *Commiphora myrrha* (T.Nees) Engl. [Burseraceae; Myrrha], *Boswellia sacra* Flück. [Burseraceae; olibanum], *Spatholobus suberectus* Dunn [Fabaceae; spatholobi caulis], *Morus alba* L. [Moraceae; cortex mori], *Achyranthes bidentata* Blume [Amaranthaceae; achyranthis bidentatae radix], *Neolitsea cassia* (L.) Kosterm. [Lauraceae; cinnamomi cortex], *Angelica sinensis* (Oliv.) Diels [Apiaceae; angelicae sinensis radix], *Pheretima aspergillum* (E. Perrier). [Megascolecidae; pheretima], *Hirudo nipponica* Whitman [Hirudinidae; hirudo], and *Buthus martensii* Karsch [Buthidae; scorpio], which have pharmacological effects of anti-inflammatory, antioxidant, and anti-arteriosclerosis (Zhang et al., 2024). The basis for taxonomic verification is Plants of the World Online (POWO, http://www.plantsoftheworldonline.org). Regulating dyslipidemia can effectively control the process of atherosclerotic cardiovascular disease (Scicchitano et al., 2014). Lu et al. used high fat diet (HFD)-fed SD rats as the model and administered 400 mg/kg/d of NXT treatment, discovering that NXT significantly reduced the serum and liver TC and TG, while reconstructing the intestinal microflora of HFD-fed SD rats, thus confirming that NXT was effective in preventing hyperlipidemic disease (Lu et al., 2022). Wang et al. constructed myocardial I/R injury by coronary artery ligation and release in C57BL6 mice, administering NXT treatment (700 mg/kg/d) before surgery. Their results showed that NXT reversed I/R injury in the heart, partly by inhibiting the activation of the NLRP3 inflammasome (Wang et al., 2016). Currently, most reports on NXT’s pharmacological effects emphasize its direct action *in vivo*, while its indirect action remains underexplored. Numerous studies on circulating exosomes as novel mediators of intercellular signaling have indicated their inducibility and regulatory potential in various diseases (Débora et al., 2022). Chemotherapy (Bandari et al., 2018), food compounds (Papini et al., 2023), and conventional drugs (Sekhavati et al., 2022) can all induce the release and regulation of exosomes. However, there are no reports or studies exploring whether NXT can stimulate exosomes release or exert regulatory effects via circulating exosomes.

Therefore, in order to investigate the effects of NXT-regulated circulating exosomes on cardiovascular and cerebrovascular diseases associated with vascular endothelial injury, circulating exosomes from SD rats orally administered with NXT (NXT-Exo) or with saline (Ctl-Exo) were extracted. Lipopolysaccharide (LPS)-treated human microvascular endothelial cells (HMEC-1) was used as models for VECs injury in this paper. The effects of NXT-Exo on HMEC-1 activity and damage were evaluated using CCK-8 and microplate methods. Enzyme linked immunosorbent assay (ELISA) and fluorescence techniques examined the impact of NXT-Exo on cell contraction and relaxation factors, inflammatory factors and oxidative stress levels. Flow cytometry and Western blot assessed the influence of NXT-Exo on cell apoptosis. RNA-seq of HMEC-1 was employed to identify differentially expressed genes (DEGs) between groups and conduct KEGG pathway enrichment analysis. RT-qPCR validated the mRNA expression levels of vascular endothelial-related differential genes. RT-qPCR and Western blot examined the influence of NXT-Exo on the TLR4/TRIF/NF-κB inflammatory pathway. Subsequently, miRNA-omics identified 12 differentially expressed miRNAs (DEMs). Integrated RNA-seq analysis further revealed miR-382-5p and *STC1* as critical factors mediating the functional effects of NXT-Exo. Functional experiments involving miR-382-5p mimics and si-STC1 transfection, combined with Western blot, confirmed the regulatory relationship between miR-382-5p and STC1. Finally, human umbilical vein endothelial cells (HUVEC) were utilized to validate the protective role of NXT-Exo against LPS-induced VECs injury. In summary, these results will provide a novel perspective for elucidating the pharmacological mechanisms by which NXT-Exo protect against endothelial injury and for the clinical application of NXT.

## 2 Materials and methods

### 2.1 Ethical statement and animals

Twenty male Sprague-Dawley (SD) rats, aged 6–8 weeks and weighing 180∼220 g, were purchased from the Laboratory Animal Center of Sun Yat-sen University. The animal production license number is SCXK (Guangdong) 2021-0029. The animal use license number is SYXK (Guangdong) 2021-0112. All experimental procedures were performed in accordance with the Guidelines for the Care and Use of Laboratory Animals published by the US National Institutes of Health and approved by the Animal Care and Use Committee of the School of Life Sciences, Sun-Yat-sen University (Approval No. SYSU-IACUC-2023-000933, Approval Date: 2023-06-05).

### 2.2 Grouping administration and circulating exosomes extraction

SD rats were randomly divided into two groups (10/group) and received oral gavage treatment for 7 days: 1. Blank control group, normal diet, saline administration (5 mL/kg/d); 2. NXT group, normal diet, NXT administration (1,000 mg/kg/d). Three NXT doses of 250, 500, and 1,000 mg/kg/d were used in the preliminary study of our team, among which 1,000 mg/kg/d had the most significant inhibitory effect on endothelial dysfunction and oxidative stress, so the 1,000 mg/kg/d NXT dose was selected for subsequent gavage (Zhang W. J. et al., 2020). NXT (Med-drug permit no. Z20025001) was obtained from Shanxi Buchang Pharmaceutical Co. Ltd. (Xianyang, China). All identification of metabolites in NXT and quality control of the preparation strictly adhered to the standards specified under “Naoxintong Capsule” in the Chinese Pharmacopoeia (2020 Edition), which were implemented by the manufacturer. All NXT used in this study were from the same batch. Treatments were given and body weights were measured once a day. At the end of the experiment, blood was drawn from the abdominal aorta of SD rats to sterile centrifuge tubes. The 10 mL blood was extracted from each SD rat and was centrifuged at 8,000 g for 30 min at 4 °C. The sediment was discarded, and the serum was further purified by centrifuging at 10,000 g for 30 min using a Thermo Multifuge X1R centrifuge at 4 °C (Thermo Scientific, United States). A total of 110 mL serum was ultra-centrifuged at 4 °C using the SW28 rotors of an OptimaL-100XP centrifuge at 140,000 g for 90 min (Beckman, United States), and the circulating exosomes were dissolved in PBS for later identification (Wei et al., 2021).

### 2.3 Identification and concentration of circulating exosomes

The morphological characters of circulating exosomes were imaged by JEM-1400 Flash electron microscopy (JEOL, Japan) with phosphotungstic acid staining. The particle size distribution of circulating exosomes was analyzed by NanoSight NS300 (Malvern Panalytical, United Kingdom). Expressions of exosome surface markers (ALIX, CD9, and TSG101) and negative control marker (β-actin) were detected by Western blot. Concentrations of circulating exosomes were measured by BCA kit (P0012, Beyotime, China). The circulating exosome PBS solution was adjusted to 1 mg/mL based on protein concentration, aliquoted, and stored at −80 °C for subsequent use.

### 2.4 Cells culture

HMEC-1 (CRL-3243) and HUVEC (PCS-100-013) were purchased from American Tissue Culture Collection (ATCC, United States). Both cell types were cultured in cell incubators at 5% CO_2_ and 37 °C using endothelial cell medium supplemented with 5% exosome-free fetal bovine serum, 1% penicillin/streptomycin, and 1% endothelial cell growth supplement (1001, ScienCell, Carlsbad, United States). The cell culture medium was replaced every 2 days, and cells reaching 80% density were passaged at a ratio of 1:3 density. HMEC-1 and HUVEC between passages 3 and 8 were used in this study.

### 2.5 Uptake of circulating exosomes by VECs

Circulating exosomes were labeled with PKH26 (MINI26-1KT, Sigma, United States). The 400 μL PKH26 (2 μM) labelling solution was mixed with 200 μL circulating exosomes for 10 min, then 1.2 mL of 1% bovine serum albumin was added and mixed, followed by centrifugation at 100,000 g for 1 h to remove PKH26 labelling solution. The exosome sediment was then resuspended in PBS. HMEC-1 and HUVEC were stained with 50 μL Dio (HY-D0969, MCE, United States) and Hoechst 33342 (S0485, Selleck, United States). Subsequently, the PKH26-labeled circulating exosomes (5 μg/mL protein) were added into 12-well plates pre-inoculated with HMEC-1 and HUVEC for 2 h incubation. The cells were then fixed with 4% paraformaldehyde. Finally, the uptakes of circulating exosomes were visualized using an LSM 880 laser confocal microscope (Zeiss, Germany).

### 2.6 Cell counting kit-8 (CCK-8) assay

The CCK-8 assay (CK04, Dojindo, Japan) was used to test cell viability. Cells in logarithmic phase were seeded in 96-well plates (3 × 10^3^ cells per well) and cultured in ECM complete medium for 12 h to allow for cell adhesion. Subsequently, the medium was replaced with ECM complete medium containing 25, 50, 100, 200, 400, and 800 ng/mL LPS for 24 h. Following incubation, 10 μL CCK-8 reagent was added to each well and incubated at 37 °C for 1 h. Cell viability was determined by measuring the absorbance at 450 nm using a microplate detector (BioTek, United States). After the concentration of LPS applied to the model group was selected, cells were inoculated as described above. After cell adhesion, the medium was replaced by the ECM complete medium containing 200 ng/mL LPS, 200 ng/mL LPS + 2.5 μg/mL Ctl-Exo, 200 ng/mL LPS + 5 μg/mL Ctl-Exo, 200 ng/mL LPS + 10 μg/mL Ctl-Exo, or 200 ng/mL LPS + 2.5 μg/mL NXT-Exo, 200 ng/mL LPS + 5 μg/mL NXT-Exo, 200 ng/mL LPS + 10 μg/mL NXT-Exo for 24 h. Following treatment, cells were incubated with 10 μL CCK-8 reagent for 1 h at 37 °C, and the absorbance value at 450 nm was measured using a microplate detector.

### 2.7 Lactate dehydrogenase (LDH) assay

The LDH concentrations of HMEC-1 and HUVEC were detected with the LDH kit (A020-2-2, Nanjingjiancheng, China) according to the manufacturer’s instructions. In the test hole, 16 μL sample solution, 20 μL matrix buffer, and 4 μL Coenzyme I solution were added successively and mixed, and the temperature bath was 37 °C for 15 min. Then 20 μL 2, 4-dinitrophenylhydrazine were added and bathed at 37 °C for 15 min, and finally 200 μL 0.4 mol/L NaOH was mixed. The absorbance value at 440 nm was measured by microplate reader.

### 2.8 Nitric oxide (NO) detection

Extracellular NO secretion levels of HMEC-1 and HUVEC were detected with the NO kit (A013-2-1, Nanjingjiancheng, China). According to the manufacturer’s instructions, 100 μL cell supernatants were collected and treated with 200 μL reagent 1 and 100 μL reagent 2 for 10 min. Following this, the mixture was centrifuged at 2,000 rcf for 15 min, and the resulting supernatants were collected. Then 160 μL supernatants were added to 80 μL chromogenic agents and allowed to react for 15 min. Subsequently, the absorbance value at 550 nm were detected by microplate reader.

### 2.9 ELISA

The expression levels of IL-1β (SEA563Hu, Cloud-clone, China), IL-6 (SEA079Hu, Cloud-clone, China), IL-8 (SEA080Hu, Cloud-clone, China), VCAM-1 (SEKH-0055, Solarbio, China), ICAM-1 (SEKH-0053, Solarbio, China), eNOS (CSB-E08322h, Cusabio, China), and ET-1 (CEA482Hu, Cloud-clone, China) were measured by quantitative ELISA kits according to the kit protocol. First, 100 μL cell supernatants from HMEC-1 and HUVEC and 100 μL standards of different concentrations were added to the 96-well ELISA plate, and incubated at 37 °C for 1 h. Subsequently, the contents were discarded, and the wells were treated with the 100 μL working solution and 90 μL TMB substrate solution for the specified reaction time. Finally, 50 μL termination solutions were added and the OD450 values were immediately measured by microplate reader.

### 2.10 Flow cytometry analysis of apoptosis

Apoptosis rates of HMEC-1 and HUVEC were detected with Annexin V, FITC apoptosis kit (AD10, Dojindo, Japan). Cells were centrifuged at 1,000 rcf for 3 min, and supernatants were discarded. Subsequently, cells were re-suspended in 500 μL 1 × Annexin V binding solution, and 100 μL suspension was incubated with 5 μL Annexin V-FITC and PI solution at 37 °C for 15 min away from light. Following this, 400 μL 1× Annexin V binding solution was added, and the samples were examined by flow cytometry. Unstained cells were used as the negative control.

### 2.11 Western blot

Cells and circulating exosomes were lysed with ice-cold RIPA lysis buffer (P0013B, Beyotime, China). Samples (10 μg) were loaded onto 10% sodium dodecyl sulfate/polyacrylamide gel electrophoresis (1610173, Bio-Rad, United States) and subsequently transferred to polyvinylidene fluoride membranes. After transfer, the membranes were blocked with sealing liquid and incubated for 2 h. Following blocking, membranes were incubated with primary antibodies against β-actin (dilution 1:1,000, ab8227, Abcam), ALIX (dilution 1:1,000, ab275377, Abcam), TSG101 (dilution 1:1,000, ab125011, Abcam), CD9 (dilution 1:1,000, ab236630, Abcam), Bax (dilution 1:1,000, ab32503, Abcam), Bcl-2 (dilution 1:1,000, ab32124, Abcam), Cleaved caspase 3 (dilution 1:500, ab32042, Abcam), TLR4 (dilution 1:1,000, TA382568, OriGene), TRIF (dilution 1:1,000, TA382523, OriGene), NF-κB p65 (dilution 1:1,000, TA385159, OriGene), Phospho-NF-κB p-p65 (dilution 1:1,000, TA380848, OriGene), STC1 (dilution 1:5000, ab229477, Abcam) at 4 °C overnight. Subsequently, the membranes were incubated with the secondary antibody horseradish peroxidase-labeled goat anti-rabbit immunoglobulin G (dilution 1:5,000, ab6721, Abcam). Immunoblots were visualized using the Tanon 5200 chemiluminescence imaging system (Tanon, China) and analyzed with the ImageJ software.

### 2.12 Reactive oxygen species (ROS) production assay

The production levels of ROS in HMEC-1 and HUVEC were determined by fluorometric assay with DCFH-DA as a probe for the presence of ROS (R252, Dojindo, Japan). Cells in logarithmic phase were seeded in 96-well black plates (3 × 10^3^ cells per well), and cultured in ECM complete medium for 12 h. After treatment, supernatants were removed and 100 μL DCFH-DA working solution was added per well. Samples were then incubated at 37 °C for 30 min. Subsequently, the DCFH-DA working solution was removed, and the cells were washed twice with PBS. Cells were detected under Spark fluorescence microplate reader (Tecan Spark, Switzerland).

### 2.13 Malondialdehyde (MDA) assay

According to the instructions of the MDA kit (M496, Dojindo, Japan), cell suspensions were collected and centrifuged, mixed with 100 μL Antioxidant PBS solution, and transferred into the labeled sample tube. The MDA standard solution with a concentration of 10 μmol/L was diluted in equal proportion to different concentrations for the preparation of the standard curve. Then, 100 μL lysis buffer and 250 μL working solution were added to sample tubes and standard tubes. After mixing, the tubes were heated in a metal bath at 95 °C for 15 min, immediately bathed in an ice bath for 5 min, cooled to room temperature, centrifuged at 10,000 g for 10 min, and 100 μL supernatant was taken and added to a black 96-well plate. The fluorescence intensity was detected with a fluorescent enzyme marker, and the parameters were set as follows: excitation wavelength was 540 nm, emission wavelength was 590 nm. The MDA concentration was calculated according to the MDA standard curve.

### 2.14 RNA-seq of HMEC-1 and miRNA-omics of circulating exosomes

After cell treatment, 1 mL TRIzol reagent was added to each hole of the 6-well plate and cracked on ice. The cell lysate was collected and stored in the frozen storage tube and stored in the refrigerator at −80 °C for sample testing. The cDNA libraries were sequenced on the Illumina sequencing platform by Genedenovo Biotechnology Co., Ltd. (Guangzhou, China). In brief, the testing process begins with sample testing, then cDNA library construction, library quality control, and finally sequencing and data processing. Ctl-Exo and NXT-Exo samples stored at −80 °C were sent for miRNA sequencing (LC-Bio Technologies, Hangzhou, China). Similarly, the first step was library construction, then sequencing, and finally data analysis. The mRNA and miRNA sequencing data of HMEC-1 have been deposited in the NCBI Gene Expression Omnibus (GEO) database and are accessible through GEO Series accession number GSE235049 and GSE298553. The threshold value for selection of DEGs and DEMs was *p*-value <0.05 and fold change (FC) ≥ 2 or ≤0.5, and the results were filtered using the False Discovery Rate (FDR) < 0.05. KEGG classifications were used to analyse DEGs.

### 2.15 Reverse transcription quantitative polymerase chain reaction (RT-qPCR)

Total RNA was extracted with EZ-press RNA Purification Kit (B0004D, EZBioscience, United States) from HMEC-1 under the guidance of the manufacturer’s protocol. Total RNA was isolated from circulating exosomes using TRIzol reagent (15596018CN, Thermo Fisher Scientific, United States). The concentration of the isolated RNA samples was measured by Nanodrop 2000 (Thermo Fisher Scientific, United States), and cDNA was obtained by reverse transcription of cells RNA with StarScript Pro reverse transcription premix (A240, Genstar, China) and by reverse transcription of circulating exosomes RNA with Synthesis of miRNA first strand cDNA (B532451-0020, Sangon Biotech, China). RT-qPCR was then performed using GoTaq qPCR Master Mix (A6002, Promega, United States), following the manufacturer’s instruction. Reaction procedures were 95 °C for 3 min, then 45 cycles at 95 °C for 10 s and 60 °C for 30 s. Primers were synthesized by Sangon (Sangon Biotech, China). The reverse primers of these four miRNAs and the primers of U6 were universal and provided in the miRNA first strand cDNA kit. Relative levels of mRNA and miRNA were normalized to β-actin and U6, and the relative expression levels of mRNA and miRNA were calculated using the 2^−ΔΔCT^ method. The primer sequences of mRNAs are shown in Table 1.

**TABLE 1:** Primer sequences of mRNAs used for RT-qPCR.

Genes	Forward sequence (5′-3′)	Reverse sequence (5′-3′)
*β-actin*	TGA​ATG​ATG​AGC​CTT​CGT​GC	CTG​GTC​TCA​AGT​CAG​TGT​AC
*IL-6*	CTC​TTC​AGA​ACG​AAT​TGA​CAA​AC	CAG​TGC​CTC​TTT​GCT​GCT​TT
*IL-8*	TCT​TGG​CAG​CCT​TCC​TGA​TT	TTT​CTG​TGT​TGG​CGC​AGT​GT
*IL6ST*	CAC​CCT​GTA​TCA​CAG​ACT​GGC​A	TTC​AGG​GCT​TCC​TGG​TCC​ATC​A
*TNFRSF9*	TGC​TTG​TGA​ATG​GGA​CGA​AGG​AGA	AGA​AAC​GGA​GCG​TGA​GGA​AGA​ACA
*IGSF8*	CCT​CGC​CAA​AGC​CTA​TGT​TCG​A	GGT​ACA​CTG​TGC​CTC​CTG​CTA​G
*BCL2*	ACT​GGC​TCT​GTC​TGA​GTA​AG	CCTGATGCTCTGGGTAAC
*BAX*	TCA​GGA​TGC​GTC​CAC​CAA​GAA​G	TGT​GTC​CAC​GGC​GGC​AAT​CAT​C
*CDH5*	TAC​CAG​GAC​GCT​TTC​ACC​AT	AAA​GGC​TGC​TGG​AAA​ATG​GG
*ATG13*	AGT​CAA​GTG​CCT​AGC​CTC​AC	GCC​TGC​TCC​AAT​CCT​CAG​AA
*ATG14*	GCG​CCA​AAT​GCG​TTC​AGA​G	AGT​CGG​CTT​AAC​CTT​TCC​TTC​T
*NF-κB*	TGA​ACC​GAA​ACT​CTG​GCA​GCT​G	CAT​CAG​CTT​GCG​AAA​AGG​AGC​C
*TLR4*	CCC​TGA​GGC​ATT​TAG​GCA​GCT​A	AGG​TAG​AGA​GGT​GGC​TTA​GGC​T
*TRIF*	CGA​CAG​TCG​AAG​TTG​GAG​GTG​A	ACC​TTC​TGC​GAG​GAT​TTC​CAG​G
*STC1*	GGA​ATC​TGT​CAT​GAG​GGG​CAA	GTG​AAA​CAG​GGA​GCG​TGT​GT

### 2.16 Construction of miRNA-mRNA network

The DEMs between Ctl-Exo and NXT-Exo groups were screened and summarized. Target genes were predicted by Miranda (v3.3a) and TargetScan (Version: 7.0) methods. TargetScan score ≥50, Miranda Energy < −10. The intersection of target gene prediction results obtained by the two methods was taken as the result of miRNAs target gene prediction. The predicted miRNAs target genes were then intersected with the DEGs in the RNA-seq of HMEC-1 to determine the differentially targeted genes of miRNAs in NXT-Exo’s anti-endothelial injury effect. CytoScape 3.10.1 software was used to map miRNA-mRNA networks to analyze and screen key miRNAs.

### 2.17 Transfection of miRNA mimics, miRNA inhibitors and si-STC1 into HMEC-1

All the miRNA mimics, miRNA inhibitors and si-STC1 used in the study were synthesized by Sangon (Sangon Biotech, China). miRNA and si-RNA transfection were performed using the RNA transfection reagent (E607402, Sangon Biotech, China) and followed the manufacturer’s direction. The final concentration of 20 μM was prepared by adding DEPC water into the miRNA and si-RNA tube. The transfection reagent was prepared by taking 380 μL ECM complete medium from tube A, adding 24 μL RNATransMate reagent and mixing well. Tube B was mixed 20 μL miRNA inhibitor, 10 μL miRNA mimic or 20 μL si-STC1 with 200 μL ECM. Tube A and tube B were mixed to make transfection mixture, and the mixture was left at room temperature for 10 min miRNA inhibitor/RNA TransMate, miRNA mimic/RNA TransMate or si-STC1/RNA TransMate complexes were formed. HMEC-1 were inoculated into 24-well plates in advance. When the cell density reached 80%, 50 μL complexes and 450 μL ECM were added into each well. The final concentration of miRNA inhibitor and si-STC1 was 50 nM, and the final concentration of miRNA mimic was 25 nM. The 24-well plates were transfected in a 37 °C incubator for 6 h, and the liquid was discarded. The group molding was performed and the drug was administered for 24 h. The preparation methods of miRNA inhibitor-NC/RNA TransMate, miRNA mimic-NC/RNA TransMate, and si-STC1-NC/RNA TransMate complexes were the same as above (Tong et al., 2023). The primer sequences of miRNAs are shown in Table 2.

**TABLE 2:** Primer sequences of miRNAs used for RT-qPCR.

miRNAs	Forward sequence (5′–3′)
miR-1-3p	CGG​CTG​GAA​TGT​AAA​GAA​GTG​TG
miR-382-5p	GAA​GTT​GTT​CGT​GGT​GGA​T
miR-802-5p	GCG​CAG​TCA​GTA​ACA​AAG​AT
miR-202-5p	GCG​CAG​TTC​CTA​TGC​ATA​TA

### 2.18 Statistical analysis

Scientific illustrations were created with BioRender. Experimental data were analyzed using Graphpad Prism 9.3.1 and expressed as the mean ± SD. The significance of the difference between two groups was assessed by Student’s t-test. Multiple group differences were compared using one-way analysis of variance (ANOVA). *p* < 0.05 was considered statistically significant.

## 3 Results

### 3.1 Isolation and characterization of circulating exosomes from NXT-administered SD rats

**FIGURE 1:**
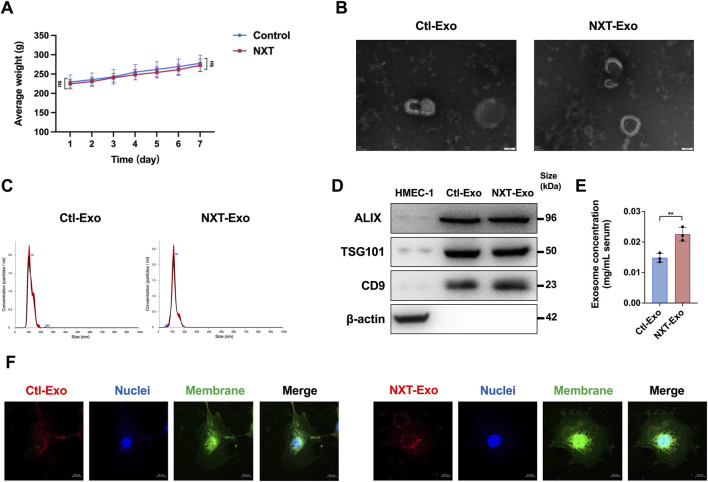
Body weight map and biological identification, concentration determination, and internalization of circulating exosomes. **(A)** Daily body weight of SD rats during the experiment. **(B)** Circulating exosomes scanning results of TEM (Scale bar, 50 nm). **(C)** The particle diameter distribution of circulating exosomes tested by NTA. **(D)** The circulating exosome markers (ALIX, TSG101, CD9) and the negative control marker (β-actin) detected by Western blot. **(E)** The concentrations of circulating exosomes tested by BCA. **(F)** The internalization of circulating exosomes from SD rats in HMEC-1 imaged by confocal microscopy (Scale bar, 10 μm). PKH-26 (Ex: 551 nm, Em: 562∼580 nm) labeled circulating exosomes, Dio (Ex: 484 nm, Em: 496∼520 nm) labeled cell membrane, and Hoechst 33342 (Ex: 346 nm, Em: 455∼470 nm) labeled cell nuclei. Values are expressed as mean ± SD, n = 3. ^*^ *p* < 0.05, ^**^ *p* < 0.01 and ns indicated no significant statistical difference.

To monitor the effects of NXT on SD rats, we daily administered SD rats and recorded their body weights. Compared with the blank control group orally administered with saline, the NXT group had no obvious weight changes, confirming that NXT did not affect the healthy growth of SD rats (Figure 1A). We then extracted NXT-Exo and Ctl-Exo, characterizing them using transmission electron microscopy (TEM), nanoparticle tracking analysis (NTA), and Western blot analysis. TEM indicated that the exosomes exhibited a typical spherical bilayer membrane structure with diameters of approximately 100 ∼ 150 nm (Figure 1B), while NTA showed that the majority of exosome particles diameter <200 nm. The results of both experiments were consistent (Figure 1C). Western blot results confirmed the presence of exosomal surface markers ALIX, CD9, and TSG101 (Figure 1D). Crucially, both NXT-Exo and Ctl-Exo exhibited typical exosome morphology and diameter distribution, while the protein concentration of NXT-Exo was significantly higher than that of Ctl-Exo, as measured by BCA (Figure 1E). This suggested that NXT stimulated the generation of circulating exosomes. Furthermore, we observed the internalization of PKH26-stained circulating exosomes by both HMEC-1, demonstrating the ability of circulating exosomes to enter VECs and potentially deliver signals (Figure 1F).

### 3.2 Protective effect of NXT-Exo on LPS-induced injury of HMEC-1

**FIGURE 2:**
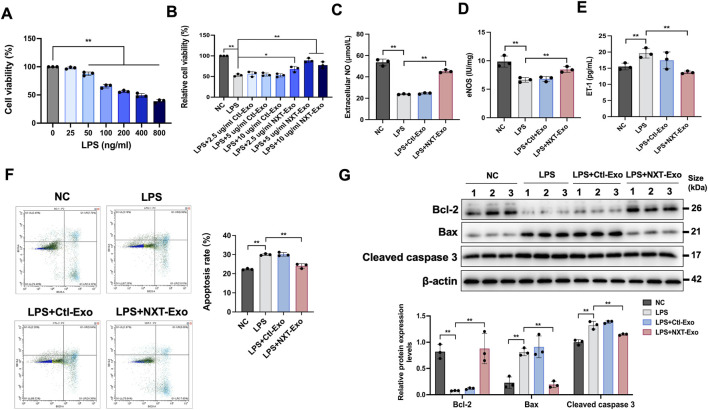
NXT-Exo inhibited LPS-induced HMEC-1 dysfunction and apoptosis. **(A)** The cell viability of HMEC-1 after LPS stimulation were detected by CCK-8 assay. **(B)** The effects of NXT-Exo on the cell activity of HMEC-1 induced by LPS were detected by CCK-8 assay. **(C–E)** The expression levels of NO, eNOS and ET-1 in HMEC-1 were measured by ELISA. **(F)** The apoptosis rates of HMEC-1 were detected by flow cytometry. The horizontal axis B525-A corresponds to the detection of Annexin V staining (Ex = 494 nm, Em = 518 nm), and the vertical axis B610-A corresponds to the detection of PI staining (Ex = 535 nm, Em = 617 nm). **(G)** The relative protein expression levels of Bax, Bcl-2 and Cleaved caspase 3 were detected by Western blot. Values are expressed as mean ± SD, n = 3. ^*^ *p* < 0.05 and ^**^ *p* < 0.01.

To investigate the protective effects of NXT-Exo against LPS-induced injury in HMEC-1, cells were exposed to increasing gradient doses of LPS for 24 h. An initial dose-response experiment revealed a decrease in cell viability in an LPS dose-dependent manner, with significant inhibition at concentrations ≥200 ng/mL (Figure 2A). We therefore selected 200 ng/mL LPS for subsequent experiments. In order to verify whether circulating exosomes can reduce the VECs injury induced by LPS, HMEC-1 were treated with Ctl-Exo or NXT-Exo in the presence of LPS. While Ctl-Exo had no significant effect on cell viability, NXT-Exo at 5 and 10 μg/mL significantly improved the cell viability in HMEC-1 compared to LPS treatment (Figure 2B). Considering the limited and valuable extractions of circulating exosomes from SD rats, a single dose of 5 μg/mL Ctl-Exo and NXT-Exo was chosen for subsequent cell experiments in order to comply with the “3R principle” (i.e., Replacement, Reduction, and Refinement) of experimental animal ethics and minimize exosome wastage.

### 3.3 NXT-Exo regulates LPS-induced relaxation and contraction factor dysregulation in HMEC-1

Regulating the dynamic balance of relaxation and contraction to maintain homeostasis is an important function of VECs (Gupta et al., 2020). To investigate the effect of NXT-Exo on endothelial tension, eNOS, NO, and ET-1 levels were measured. The results showed that compared with the NC group, the expression of eNOS and NO in the LPS group was downregulated, and the expression of ET-1 was upregulated. The NO elevation may be associated with enhanced eNOS activity. NXT-Exo significantly reversed the LPS-induced downregulation of NO (Figure 2C) and eNOS (Figure 2D) and upregulation of ET-1 (Figure 2E), whereas Ctl-Exo had no significant effect.

### 3.4 NXT-Exo mitigates LPS-induced apoptosis in HMEC-1

To further evaluate the protective effect of NXT-Exo against LPS-induced apoptosis, we measured cell apoptosis rates using flow cytometry. Results indicated that NXT-Exo significantly reduced LPS-induced apoptosis (Figure 2F). Additionally, Western blot analysis revealed that NXT-Exo markedly attenuated LPS-induced upregulation of Bax and Cleaved caspase 3 and downregulation of Bcl-2 in HMEC-1 (Figure 2G)**.**

### 3.5 NXT-Exo inhibits LPS-induced inflammation and oxidative damage in HMEC-1

**FIGURE 3:**
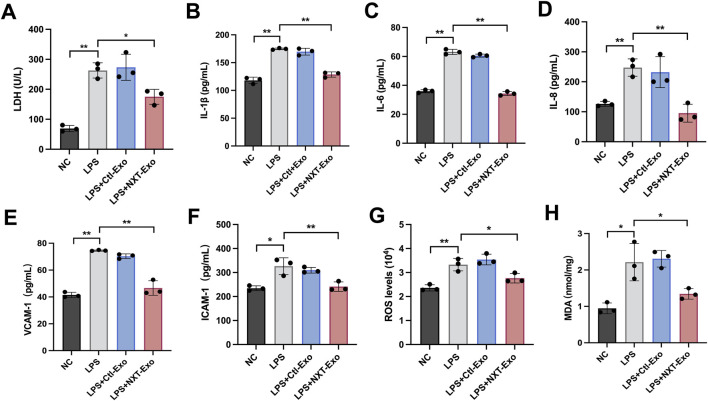
NXT-Exo protected HMEC-1 from LPS-induced inflammation, and oxidative damage. **(A)** LDH releases from HMEC-1 was detected by microplate method. **(B–F)** The secretion levels of IL-1β, IL-6, IL-8, VCAM-1 and ICAM-1 were measured via ELISA. **(G,H)** The expression levels of ROS and MDA were detected by fluorescence method. Values are expressed as mean ± SD, n = 3. *p < 0.05 and **p < 0.01.

To assess the extent of cell damage, we employed the LDH assay and observed that, compared with the LPS group, Ctl-Exo treatment exhibited no significant change in LDH level, whereas NXT-Exo treatment significantly reduced LDH level (Figure 3A). These results suggested that NXT-Exo could possess the capability to mitigate LPS-induced VECs injury. Given that inflammation-mediated VECs damage greatly increases the risk of cardiovascular and cerebrovascular diseases, we examined inflammatory factors using ELISA assay. Our findings demonstrated that the secretion levels of IL-1β, IL-6, IL-8, and adhesion molecules VCAM-1, ICAM-1 were notably increased following LPS challenge. However, treatment with NXT-Exo effectively inhibited the LPS-induced elevation of these factors (Figures 3B–F). Moreover, considering that LPS-induced ROS generation is a major contributor to cytotoxicity, we assessed the ROS fluorescence intensity and MDA concentration. Our results revealed a significant increase in ROS and MDA levels in the LPS group, while NXT-Exo treatment considerably alleviated these markers (Figures 3G,H).

### 3.6 RNA-seq reveals potential mechanisms of NXT-Exo protection against LPS-induced HMEC-1 injury

**FIGURE 4:**
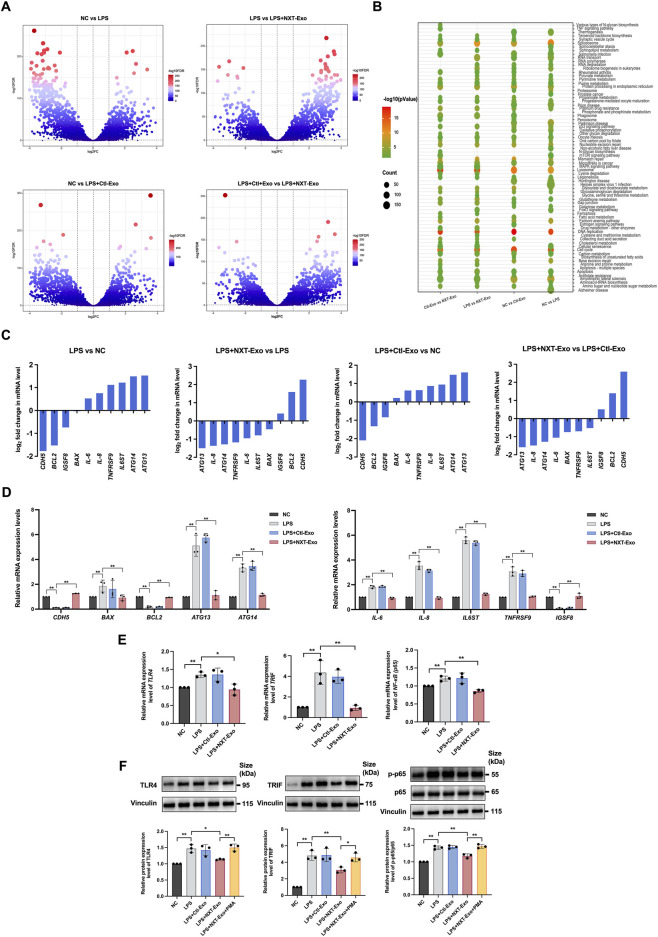
RNA-seq of HMEC-1. **(A)** Volcano plots of DEGs (n = 5). **(B)** KEGG enrichment bubble plots of DEGs (n = 5). **(C)** DEGs related to vascular endothelial injury. **(D)** The relative mRNA expression levels of vascular endothelial injury-related genes verified by RT-qPCR (n = 3). **(E)** The relative mRNA expression levels of *TLR4, TRIF* and *p65* were detected by RT-qPCR (n = 3). **(F)** The expression protein levels of TLR4, TRIF and p-p65/p65 were detected by Western blot (n = 3). Values are expressed as mean ± SD. *p < 0.05, **p < 0.01. The screening criteria for DEGs were defined as FC ≥ 2 or ≤0.5, FDR < 0.05 and *p*-value <0.05.

To identify the molecular mechanisms underlying NXT-Exo’s protective effects against LPS-induced VECs injury, we performed RNA-seq analysis on HMEC-1, using FC ≥ 2 or ≤0.5, FDR < 0.05 and *p*-value <0.05 as the screening criteria. We analyzed DEGs between the following groups: NC vs. LPS, LPS vs. LPS + NXT-Exo, NC vs. LPS + Ctl-Exo, and LPS + Ctl-Exo vs. LPS + NXT-Exo. As anticipated, numerous genes were differentially expressed in LPS compared to NC, with more genes were upregulated in LPS. Interestingly, this imbalance was mitigated in the LPS + NXT-Exo group, suggesting a protective effect of NXT-Exo against LPS injury. In contrast, the LPS + Ctl-Exo group had little effect in reversing the LPS-induced gene upregulation (Figure 4A). We then conducted KEGG enrichment analysis of DEGs between groups, highlighting the top pathways involved in DNA replication, lysosome, cell cycle, protein processing in endoplasmic reticulum and apoptosis (Figure 4B). These pathways mirrored the potential mechanisms of NXT-Exo’s protection against LPS-induced cell injury. Based on the previous findings and relevant literature, we selected ten representative genes related to vascular endothelial injury, including *CDH5*, *BAX*, *BCL2*, *ATG13*, *ATG14*, *IL-6*, *IL-8*, *IL6ST*, *TNFRSF9*, and *IGSF8* (Figure 4C), and confirmed their differential expression by RT-qPCR (Figure 4D). The RT-qPCR validation of these key genes strengthened our confidence in the RNA-seq findings and provided specific targets for further mechanistic investigation.

### 3.7 NXT-Exo inhibits the TLR4/TRIF/NF-κB inflammatory pathway against LPS-induced HMEC-1 injury

*In vivo*, LPS can be recognized and bound by lipopolysaccharide binding protein (LBP) due to its high affinity with lipid A structure. Subsequently, LPS forms a complex with CD14 under the action of lipid shuttle and presentation by LBP, resulting the formation of LPS-LBP-CD14 complex (Mattingly et al., 2022). This complex is recognized by TLR4 receptor on the VECs membrane through the PAPM mode, leading to vascular endothelial inflammation and subsequent oxidative and apoptotic damage (Marongiu et al., 2019). Studies have shown that active metabolites of natural herbal extracts (Fu et al., 2020), Chinese herbal compound preparations (Miao et al., 2022), intestinal microbiota (Li et al., 2024), and specific nucleotides (Cai et al., 2022) inhibited LPS-induced inflammation by regulating TLR4/TRIF/NF-κB or TLR4/NF-κB pathways. Based on these findings, we began to elucidate the pathway through which NXT-Exo mitigated cell injury. Consistent with this pathway, our RNA-seq analysis revealed that DEGs were related to inflammation and apoptosis, and KEGG enrichment analysis highlighted biological processes including cell cycle, lysosome, and apoptosis, which were closely related to inflammation. These results suggested that NXT-Exo may be involved in the TLR4/TRIF/NF-κB or TLR4/NF-κB pathway in ameliorating LPS-induced cell injury. To test our hypothesis, we examined the expression of *TLR4*, *TRIF*, and *p65* using RNA-seq and confirmed them by RT-qPCR. Remarkably, their expression levels were significantly increased in the LPS group, but decreased after NXT-Exo treatment (Figure 4E). Western blot assay further validated these findings at the protein level, showing increased expression of TLR4, TRIF, and p-p65/p65 ratio in the LPS group, all of which were reversed by NXT-Exo treatment. Therefore, the addition of the NF-κB activator PMA attenuated the inhibitory effects of LPS + NXT-Exo on TLR4, TRIF, and p-p65/p65 (Figure 4F), suggesting that NXT-Exo may exert NXT pharmacological effects by suppressing the TLR4/TRIF/NF-κB signaling pathway.

### 3.8 Identification and functional analysis of miRNAs with protective effects in NXT-Exo

**FIGURE 5:**
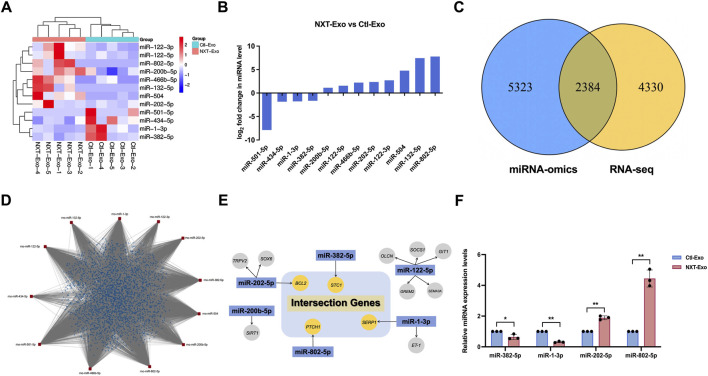
Screening of core miRNAs using circulating exosomal miRNA profiling. **(A)** Hierarchical clustering heatmap of top 12 upregulated and downregulated miRNAs (n = 5). **(B)** Map of DEMs. **(C)** Intersection Venn diagram of predicted target genes of miRNA profiling and DEGs of RNA-seq. **(D)** miRNA-mRNA network map of 12 DEMs and intersection genes. **(E)** Relationship diagram between DEMs target genes and intersection genes. **(F)** Relative miRNA expression levels of core miRNAs verified by RT-qPCR (n = 3). Values are expressed as mean ± SD. *p < 0.05, **p < 0.01. The screening criteria for DEMs were defined as FC ≥ 2 or ≤0.5, FDR < 0.05 and *p*-value <0.05.

To investigate whether NXT-Exo can inhibit LPS-induced HMEC-1 damage through miRNA, we performed miRNA-omics analysis of Ctl-Exo and NXT-Exo, using FC ≥ 2 or ≤0.5, FDR <0.05 and *p*-value <0.05 as the screening criteria. A total of 12 DEMs were screened out, hierarchical clustering heatmap shows the 12 increased and decreased miRNAs (Figure 5A), including eight upregulated miRNAs and four downregulated miRNAs (Figure 5B). Miranda (v3.3a) and Target Scan (Version: 7.0) were used to predict the target genes of these DEMs in Ctl-Exo and NXT-Exo, with screening criteria of Target Scan score ≥50 and Miranda energy < −10. The intersection of the two sets revealed 7,707 target genes. In order to ensure the reliability, the predicted target genes of miRNAs were intersected with the DEGs of RNA-seq, resulting in 2,384 overlapped genes (Figure 5C). The miRNA-mRNA network map was constructed based on these overlapped genes, which showed that all the 12 miRNAs could target the overlapped genes (Figure 5D).

Through literature analysis, we found that six out of the 12 DEMs have been reported to be associated with vascular endothelial injury or cardiovascular and cerebrovascular diseases. By comparing the target genes mentioned above with the overlapped genes, we identified miR-202-5p, miR-382-5p, miR-1-3p, and miR-802-5p as core miRNAs (Figure 5E) and verified their differential expression by RT-qPCR. The result of RT-qPCR was consistent with miRNA-omics analysis (Figure 5F).

### 3.9 The role of four core miRNAs in the inhibition of LPS-induced LDH, IL-6, and ROS expression in HMEC-1 by NXT-Exo

**FIGURE 6:**
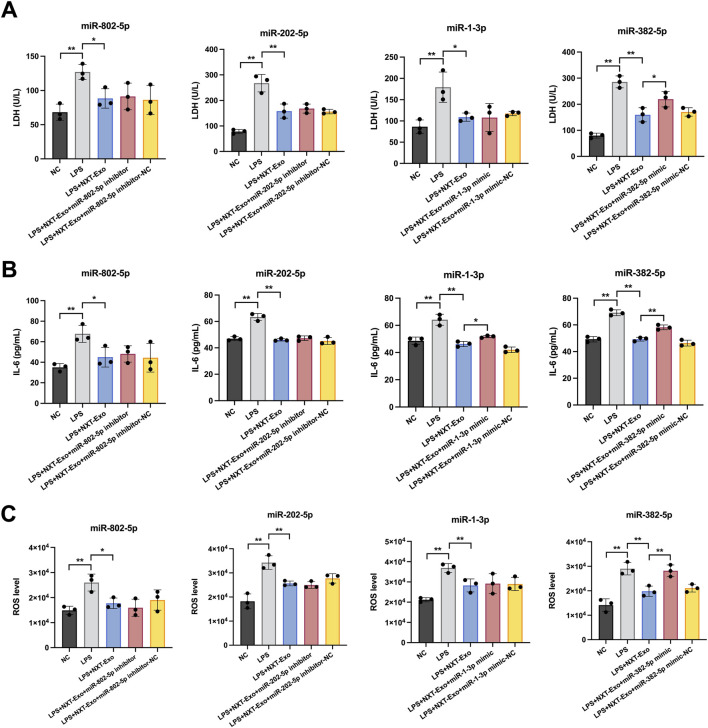
miR-382-5p was a key factor for NXT-Exo to inhibit the damage, inflammation and oxidation of HMEC-1. **(A)** The effects of core miRNAs on NXT-Exo inhibiting LPS-induced LDH releases in HMEC-1. **(B)** The effects of core miRNAs on NXT-Exo inhibiting LPS-induced IL-6 secretion levels in HMEC-1. **(C)** The effects of core miRNAs on NXT-Exo inhibiting LPS-induced ROS production levels in HMEC-1. Values are expressed as mean ± SD, n = 3. *p < 0.05, **p < 0.01.

To further study the protective effects of miRNAs, we inhibited or increased the core miRNAs expression in HMEC-1 and investigated three aspects of cell damage, including LDH release, IL-6 secretion and ROS production. Compared with the LPS + NXT-Exo group, the LPS + NXT-Exo + miR-382-5p mimic group showed a significant increase in LDH release, IL-6 secretion, and ROS production. Additionally, the LPS + NXT-Exo + miR-1-3p mimic group showed an increase in IL-6 secretion. In contrast, the LPS + NXT-Exo + miR-202-5p inhibitor group and LPS + NXT-Exo + miR-802-5p inhibitor group showed no significant changes. These results suggested that miR-382-5p played a key role in the protective effects of NXT-Exo against LPS-induced inflammatory oxidative damage in HMEC-1 (Figures 6A–C).

### 3.10 miR-382-5p downregulates *STC1* expression

**FIGURE 7:**
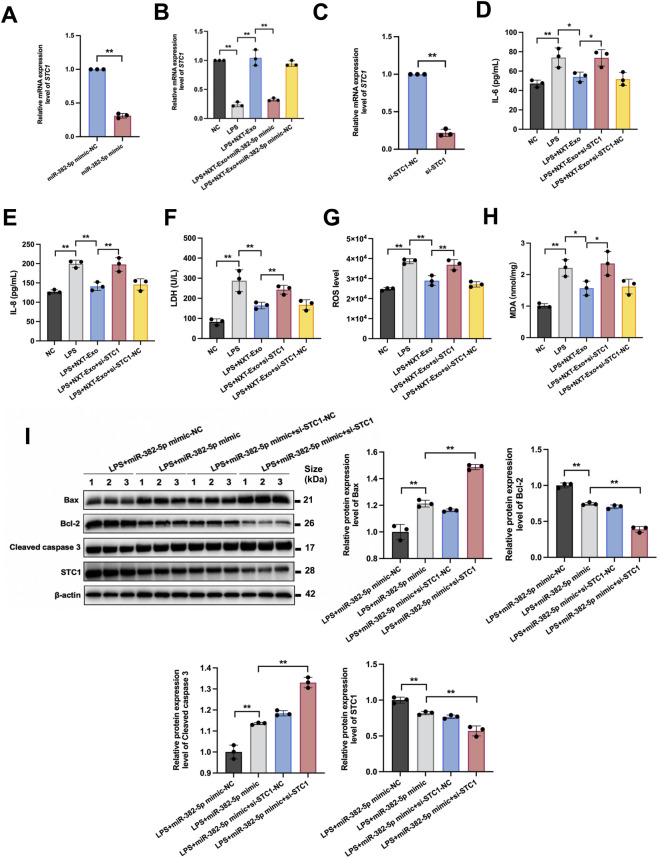
miR-382-5p downregulated the expression of STC1 and the effect of STC1 on the endothelial protection of NXT-Exo. **(A)** The mRNA expression level of STC1 in HMEC-1 transfected with miR-382-5p mimic or miR-382-5p mimic-NC was detected by RT-qPCR. **(B)** The mRNA expression level of STC1 in HMEC-1 treated with NXT-Exo or NXT-Exo combined with miR-382-5p was detected by RT-qPCR. **(C)** The transfection of si-STC1 was verified by RT-qPCR. **(D,E)** The secretion levels of IL-6 and IL-8 were measured by ELISA. **(F)** The release of LDH was detected by microplate method. **(G,H)** The production level of ROS and expression level of MDA were tested by fluorescence. **(I)** The effects of miR-382-5p and STC1 treatment on the relative protein expression levels of Bax, Bcl-2, Cleaved caspase 3 and STC1 in HMEC-1 were detected by Western blot. Values are expressed as mean ± SD, n = 3. ^*^ *p* < 0.05, ^**^ *p* < 0.01.

Based on our integrative studies above, we hypothesized that *STC1* may be regulated by miR-382-5p and contribute to NXT-Exo’s protection against LPS-induced HMEC-1 damage. Stanniocalcin-1 (*STC1*), a 35 kD homologous disaccharide protein hormone, is expressed in various cells such as neurons, megakaryocytes, and endothelial cells, possessing antioxidant and anti-inflammatory properties (Shi et al., 2018). *STC1* has been confirmed as a target of miR-382-5p, and it is involved in regulating cardiomyocyte apoptosis following acute myocardial infarction. To verify whether miR-382-5p affects the expression of *STC1*, we transfected HMEC-1 with miR-382-5p mimic or miR-382-5p mimic-NC, and detected the expression of *STC1* using RT-qPCR. The results showed that miR-382-5p mimic significantly inhibited the expression of *STC1*, suggesting *STC1* may be the target of miR-382-5p (Figure 7A).

Additionally, we co-treated HMEC-1 with NXT-Exo and either miR-382-5p mimic or miR-382-5p mimic-NC. Compared with the LPS group, NXT-Exo treatment upregulated *STC1* expression. Transfection with miR-382-5p mimic in the presence of NXT-Exo led to downregulation of *STC1* expression compared with the LPS + NXT-Exo group, indicating that miR-382-5p could reverse the promoting effect of NXT-Exo on *STC1* expression. This trend of *STC1* expression was consistent with the results of RNA-seq (Figure 7B). In conclusion, miR-382-5p downregulated the expression of *STC1*, suggesting that the protective effects of NXT-Exo against LPS-induced HMEC-1 damage involved the modulation of *STC1* through miR-382-5p.

### 3.11 Knocking down STC1 inhibited the protective effect of NXT-Exo on LPS-induced HMEC-1 damage

As miR-382-5p could inhibit the expression of *STC1*, we further explored whether *STC1* was responsible for the protective effect of NXT-Exo. We transfected HMEC-1 with si-STC1 and verified *STC1* expression by RT-qPCR. The expression level of *STC1* mRNA significantly decreased after transfection with si-STC1 compared to si-STC1-NC, indicating successful transfection and effective inhibition of *STC1* expression (Figure 7C). Next, we examined the effect of *STC1* on various cell damage markers, including LDH, IL-6, IL-8, ROS, and MDA. The results showed that si-STC1 counteracted NXT-Exo’s effect on the downregulation of these markers (Figures 7D–H), suggesting that STC1 exerted anti-inflammatory and antioxidant effects, inhibiting cell damage. Thus, STC1 might be a key molecule mediating the protective effects of NXT-Exo against LPS-induced cell injury.

### 3.12 Knocking down STC1 further exacerbated the promoting effect of miR-382-5p on LPS-induced HMEC-1 apoptosis

To determine the protective effects of miR-382-5p and STC1 on VECs apoptosis, we transfected HMEC-1 with si-STC1 and miR-382-5p mimic. Western blot results revealed that transfection with miR-382-5p mimic resulted in upregulated levels of pro-apoptotic markers Bax and Cleaved caspase 3, and downregulated levels of anti-apoptotic markers Bcl-2 and STC1. Furthermore, co-transfection with miR-382-5p mimic and si-STC1 significantly increased the expression levels of Bax and Cleaved caspase 3, while decreasing the expression levels of Bcl-2 and STC1 compared to cells transfected with miR-382-5p mimic alone (Figure 7I). These results indicated that miR-382-5p promoted LPS-induced apoptosis of HMEC-1 by down-regulating STC1, emphasizing the role of the miR-382-5p/STC1 axis in VECs apoptosis.

### 3.13 NXT-Exo inhibits LPS-induced HUVEC injury

**FIGURE 8:**
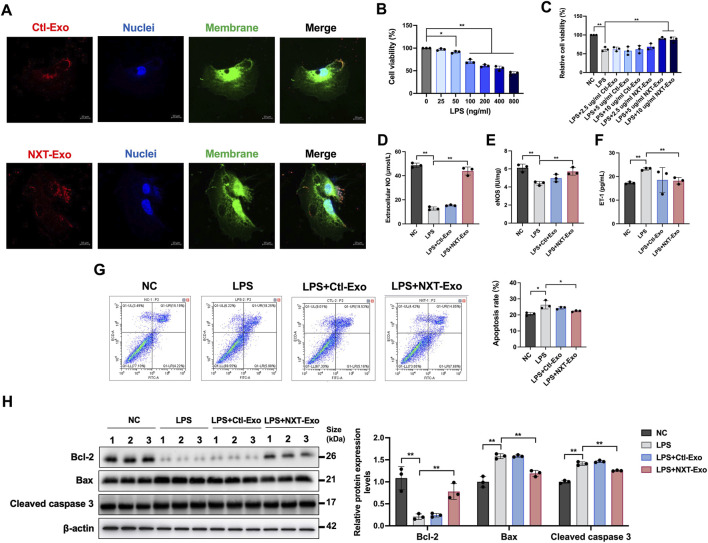
NXT-Exo inhibited LPS-induced HUVEC dysfunction and apoptosis. **(A)** The internalization of circulating exosomes from SD rats in HUVEC imaged by confocal microscopy (Scale bar, 10 μm). PKH-26 (Ex: 551 nm, Em: 562∼580 nm) labeled circulating exosomes, Dio (Ex: 484 nm, Em: 496∼520 nm) labeled cell membrane, and Hoechst 33342 (Ex: 346 nm, Em: 455∼470 nm) labeled cell nuclei. **(B)** The cell viability of HUVEC after LPS stimulation were detected by CCK-8 assay. **(C)** The effects of NXT-Exo on the cell activity of HUVEC induced by LPS were detected by CCK-8 assay. **(D–F)** The expression levels of NO, eNOS and ET-1 in HUVEC were measured by ELISA. **(G)** The apoptosis rates of HUVEC were detected by flow cytometry. The horizontal axis B525-A corresponds to the detection of Annexin V staining (Ex = 494 nm, Em = 518 nm), and the vertical axis B610-A corresponds to the detection of PI staining (Ex = 535 nm, Em = 617 nm). **(H)** The relative protein expression levels of Bax, Bcl-2 and Cleaved caspase 3 were detected by Western blot. Values are expressed as mean ± SD, n = 3. ^*^ *p* < 0.05, ^**^ *p* < 0.01.

**FIGURE 9:**
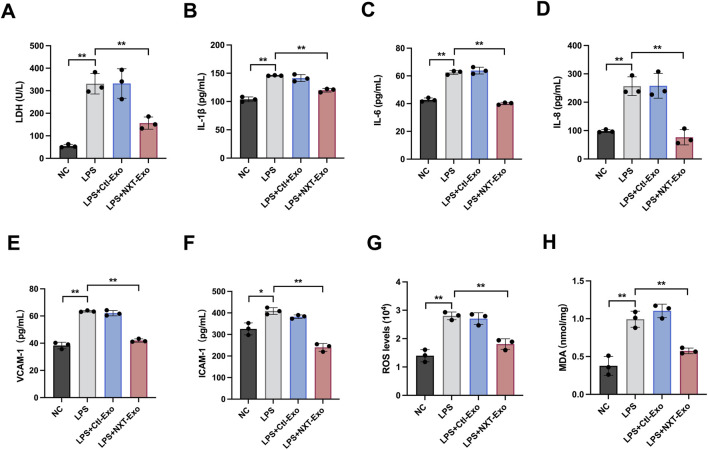
NXT-Exo protected HUVEC from LPS-induced inflammation, and oxidative damage. **(A)** LDH releases from HUVEC was detected by microplate method. **(B–F)** The secretion levels of IL-1β, IL-6, IL-8, VCAM-1 and ICAM-1 were measured via ELISA. **(G,H)** The expression levels of ROS and MDA were detected by fluorescence method. Values are expressed as mean ± SD, n = 3. *p < 0.05, **p < 0.01.

To validate the effects of NXT-Exo on vascular endothelial cells, we employed primary HUVEC for supplementary verification. Initial fluorescence experiments confirmed HUVEC uptake of PKH26-labeled circulating exosomes (Figure 8A). Subsequently, establishment of LPS and exosome concentration gradients revealed that LPS dose-dependently reduced HUVEC viability, with significant inhibition observed at concentrations ≥200 ng/mL, thus establishing 200 ng/mL as the optimal LPS concentration for subsequent experiments (Figure 8B). NXT-Exo at 5 and 10 μg/mL significantly enhanced cell survival rates in LPS-treated HUVEC, whereas Ctl-Exo showed no protective effects. The maximal protective effect of NXT-Exo was observed at 5 μg/mL, so 5 μg/mL was selected as the concentration for both Ctl-Exo and NXT-Exo in subsequent experiments (Figure 8C). We then examined NXT-Exo’s impacts on endothelial dysfunction and apoptosis. NXT-Exo effectively reversed LPS-induced downregulation of NO (Figure 8D) and eNOS (Figure 8E), while mitigating ET-1 upregulation in HUVEC (Figure 8F). Furthermore, NXT-Exo reduced LPS-induced apoptosis (Figure 8G). Western blot analysis demonstrated that NXT-Exo significantly attenuated LPS-induced upregulation of Bax and Cleaved caspase 3, along with restoration of Bcl-2 expression in HUVEC (Figure 8H), collectively indicating NXT-Exo’s protective role against LPS-induced endothelial dysfunction and apoptosis. Moreover, we investigated NXT-Exo’s effects on injury and inflammatory oxidative responses post-LPS challenge. The results showed that LPS treatment elevated LDH, IL-6, IL-1β, IL-8, VCAM-1, and ICAM-1 levels, which were effectively counteracted by NXT-Exo intervention (Figures 9A–F). Oxidative stress evaluation revealed that NXT-Exo significantly suppressed LPS-induced increases in ROS production and MDA activity in HUVEC (Figures 9G,H), suggesting NXT-Exo’s capacity to restore anti-inflammatory and antioxidant activities following LPS-induced damage. These findings aligned with the HMEC-1 experimental outcomes, further suggesting that circulating exosomes induced by NXT may confer partial protection against endothelial injury.

## 4 Discussion

NXT, a classical Chinese medicinal compound preparation, demonstrates multi-target regulatory effects on vascular function through its complex phytochemical composition (Yan et al., 2021). Pharmacological analyses identify organic acids, flavonoids, phthalides, and saponins as its primary bioactive constituents, which collectively suppress VECs inflammation and enhance vasoactive substance expression, thereby improving hemodynamic parameters and resolving blood stasis (Li W. X. et al., 2021). Preclinical evidence substantiates its therapeutic potential: In a HFD-fed Bama minipigs model, Zhang et al. reported that NXT administration (110 mg/kg/d) significantly attenuated dyslipidemia, mitigated vascular inflammation, augmented antioxidant defenses, and reduced myocardial injury, establishing its cardioprotective efficacy. It is worth noting that NXT administration had no damage to the liver and kidneys, and presented almost no side effects (Zhang et al., 2019). Mechanistic research further elucidate its vascular protective actions. *In vitro* experiments using EA. hy926 cells subjected to oxygen-glucose deprivation/reperfusion (OGD/R) revealed that 1 mg/L NXT ameliorated hypoxic-ischemic damage and prevented thrombotic cascades via modulation of the COX2-VEGF/NFκB signaling axis (Wang Z. et al., 2021). Notably, TCM has an innovative therapeutic mechanism in disease intervention by targeting and regulating cell communication mediated by circulating exosome. For instance, Ma et al. demonstrated that Qingxin Jieyu Fang-induced circulating exosomes inhibited pyroptosis in H9C2 cardiomyocytes and alleviated OGD/R injury by regulating the NLRP3/Caspase-1/GSDMD signaling pathway (Ma, 2023). In this study, we found that NXT effectively regulated the circulating exosome contents in SD rats to protect VECs from apoptosis, inflammation, oxidation, and functional destruction. Through integrative bioinformatics and functional validation, we further identified miR-382-5p and *STC1* as potential mediators of NXT-Exo pharmacological activity.

Extensive experimental evidence confirmed that LPS served as a canonical inducer of vascular endothelial inflammation and injury. LPS exposure upregulated pro-inflammatory cytokines like TNF-α, IL-1β and IL-6 in VECs (Zhang F. M. et al., 2020), concomitant with exacerbated oxidative stress and apoptotic activation (You et al., 2021). Mechanistically, LPS binds to TLR4 on VEC membranes, initiating the TLR4/TRIF signaling cascade. This triggers IκB kinase complex activation, resulting in sequential phosphorylation, ubiquitination, and proteasomal degradation of IκB (Li Y. P. et al., 2021). Subsequent NF-κB p65 translocates from the cytoplasm to the nucleus, binding to target genes and producing inflammatory cytokines such as IL-1β and IL-6, thus triggering inflammation and inducing apoptosis (Chen et al., 2024). Zhou et al. demonstrated that TLR4/NF-κB blockade effectively alleviated LPS-induced HUVEC inflammation and apoptosis, manifested by downregulation of inflammatory factors IL-1β and IL-6, upregulation of apoptotic protein Bcl-2, and downregulation of Bax and Cleaved caspase 3 (Zhou et al., 2018). Shu et al. reported that andrographolide ameliorated endothelial dysfunction through modulation of PPAR and NF-κB signaling, restoring ET-1/NO homeostasis (Shu et al., 2020). Furthermore, sustained TLR4 activation and NF-κB nuclear translocation disrupted eNOS/iNOS equilibrium, compromising endothelial vasoregulatory capacity (Gliozzi et al., 2019). Critically, these pathomechanistic findings were consistent with our RNA-seq and Western blot data, which collectively indicated that NXT-mediated regulation of circulating exosomes mitigated vascular endothelial injury through inhibition of the TLR4/TRIF/NF-κB signaling pathway.

Exosomal regulatory molecules, particularly miRNAs, serve as master regulators of intercellular signaling networks. These endogenous single-stranded non-coding RNAs mediate systemic cross-tissue communication through paracrine/endocrine mechanisms, exerting pleiotropic effects on post-transcriptional gene regulation and distal cellular functionality (Chang et al., 2021). It has been reported that numerous human pathophysiological states are related to miRNAs, highlighting their potential as targets for the diagnosis, prevention and treatment of cardiovascular and cerebrovascular diseases (Isaac et al., 2021). miR-802-5p silencing ameliorated myocardial infarction-induced damage via *PTCH1*-dependent pathways (Li S. H. et al., 2021). miR-382-5p inhibition attenuated post-AMI cardiomyocyte apoptosis through *STC1* upregulation (Zhang et al., 2021). miR-202-5p overexpression exacerbated atherosclerotic plaque instability by inducing macrophage apoptosis via inflammatory factor release and *Bcl-2* suppression (Xu et al., 2023). miR-1-3p drived endothelial dysfunction in acute lung injury by targeting *SERP1* to impair proliferation/apoptosis balance and vascular integrity (Gao et al., 2021). Through integrated miRNA-omics and RNA-seq screening, we identified four core miRNAs (miR-1-3p, miR-382-5p, miR-802-5p, miR-202-5p) within NXT-Exo. Interestingly, transfection experiments revealed that only overexpression of miR-382-5p uniquely eliminated the protective effect of NXT-Exo on HMEC-1, which can be demonstrated by restoring LDH release, IL-6 production, and ROS production. This establishes miR-382-5p as a key mediator in NXT-Exo vascular endothelial protective pharmacology.

Existing evidence delineated miR-382-5p and *STC1* as pivotal molecular determinants in cardiovascular pathophysiology. Shi et al. reported that miR-382-5p downregulation aggravated atherosclerosis progression (Shi et al., 2021). Liu et al. found that perivascular adipose-derived exosomes reduced the formation of macrophages and foam cells in atherosclerosis by upregulating miR-382-5p (Liu et al., 2022). Zhang et al. verified through a dual-luciferase reporter assay that *STC1* is a direct target of miR-382-5p and further found that interference with miR-382-5p reduced cardiomyocyte apoptosis by upregulating *STC1* (Zhang et al., 2021). Widely distributed in mammalian tissues, *STC1* plays various functions as a paracrine and autocrine factor, participating in cell metastasis, regulating cell cycle and apoptosis, inhibiting cell hypoxia and hypertonic stress damage, and promoting angiogenesis, thus behaving an important role in tumors, cardiovascular diseases, and neurological disorders (Wang P. et al., 2021). Furthermore, *STC1* as an angiogenic factor, reportedly protects against chronic heart failure post-myocardial infarction (Watanabe et al., 2021). Shi et al. demonstrated that *STC1* attenuated ROS production and NF-κB activation in inflammatory pathways, while downregulating the expression of adhesion molecules (Shi et al., 2018). Our integrative experimental validation, including miRNA-omics, ELISA, and Western blot established a mechanistic convergence wherein NXT-Exo ameliorated HMEC-1 inflammatory-oxidative injury through coordinated miR-382-5p/*STC1* axis. These experimental results suggested that *STC1* may function as a negative regulator of TLR4/TRIF/NF-κB signaling, and that NXT-Exo, by downregulating miR-382-5p, relieved the inhibition on *STC1*, thereby restraining excessive activation of the NF-κB pathway. The miR-382-5p/*STC1* axis may act upstream or parallel to the TLR4/TRIF/NF-κB pathway to mediate the anti-inflammatory and anti-apoptotic effects of NXT-Exo.

Taken together, this study established that NXT-Exo conferred robust protection against LPS-induced vascular endothelial injury through multi-modal suppression of apoptotic, inflammatory, and oxidative stress cascades. Mechanistically, this cytoprotective efficacy was mediated through the suppression of the TLR4/TRIF/NF-κB signaling pathway and the regulation of miRNA expression, particularly exosomal miR-382-5p and its target *STC1*. These findings provided novel insights into the molecular mechanisms of NXT-Exo and highlighted their potential therapeutic applications in treating vascular inflammatory diseases. However, HUVEC model was used to validate the efficacy of NXT, *in vivo* pharmacological characteristics of NXT-Exo remains uncharacterized. Furthermore, the relationship between NXT’s phytochemical metabolites and circulating exosomal miRNA cargo specificity awaits systematic phytomics-exosomeomics correlation analysis. Additionally, we will give priority to the use of atherosclerosis susceptible animal models for preclinical validation to evaluate the protective effect of NXT-Exo on vascular endothelium. Finally, further clinical cohort analysis will be conducted to characterize the dynamic changes in serum exosomal miR-382-5p/*STC1* levels after NXT administration.

## 5 Conclusion

**FIGURE 10:**
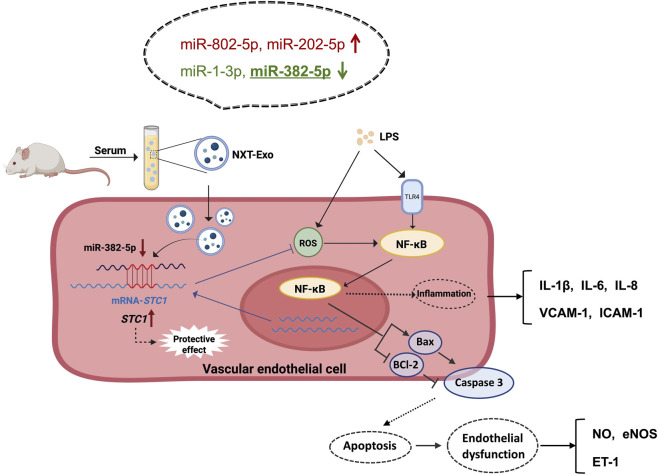
Schematic diagram illustrated that Naoxintong Capsule protected vascular endothelial cell from injury and dysfunction by downregulating miR-382-5p in circulating exosomes, potentially via targeting *STC1* and inhibiting the TLR4/TRIF/NF-κB pathway.

In conclusion, our results provided the pioneering evidence that NXT mediated downregulation of miR-382-5p in circulating exosomes, thereby safeguarding HMEC-1 from damage by targeting *STC1*, and NXT-Exo inhibited the TLR4/TRIF/NF-κB signaling pathway (Figure 10). The results of this study further complemented the mechanism of vascular endothelial injury induced by LPS and the paracrine manner protective effect of NXT, which will provide pharmacological evidence for the clinical application of NXT.

## Data Availability

The mRNA and miRNA sequencing data have been deposited in the NCBI Gene Expression Omnibus (GEO) database and are accessible through GEO Series accession number GSE235049 and GSE298553.

## References

[B1] Augustin H. G. Koh G. Y. (2024). A systems view of the vascular endothelium in health and disease. Cell 187, 4833–4858. 10.1016/j.cell.2024.07.012 39241746

[B2] Bandari S. K. Purushothaman A. Ramani V. C. Brinkley G. J. Chandrashekar D. S. Varambally S. (2018). Chemotherapy induces secretion of exosomes loaded with heparanase that degrades extracellular matrix and impacts tumor and host cell behavior. Matrix Biol. 65, 104–118. 10.1016/j.matbio.2017.09.001 28888912 PMC5816689

[B3] Bloom S. I. Islam M. T. Lesniewski L. A. Donato A. J. (2023). Mechanisms and consequences of endothelial cell senescence. Nat. Rev. Cardiol. 20, 38–51. 10.1038/s41569-022-00739-0 35853997 PMC10026597

[B4] Cai T. C. Xu L. M. Xia D. C. Zhu L. J. Lin Y. H. Yu S. J. (2022). Polyguanine alleviated autoimmune hepatitis through regulation of macrophage receptor with collagenous structure and TLR4-TRIF-NF-kappaB signalling. J. Cell. Mol. Med. 26, 5690–5701. 10.1111/jcmm.17599 36282897 PMC9667514

[B5] Chang S. N. Chen J. J. Wu J. H. Chung Y. T. Chen J. W. Chiu C. H. (2021). Association between exosomal miRNAs and coronary artery disease by next-generation sequencing. Cells 11, 98. 10.3390/cells11010098 35011660 PMC8750494

[B6] Chen Y. Li Y. Li X. J. Fang Q. Q. Li F. Chen S. Y. (2024). Indole-3-Propionic acid alleviates intestinal epithelial cell injury *via* regulation of the TLR4/NF-κB pathway to improve intestinal barrier function. Mol. Med. Rep. 30, 189. 10.3892/mmr.2024.13313 39219265 PMC11350629

[B7] Débora F. Nuno M. J. R R. L. (2022). New advances in exosome-based targeted drug delivery systems. Crit. Rev. Oncol.Hematol. 172, 103628. 10.1016/j.critrevonc.2022.103628 35189326

[B8] Dong X. Gao X. J. Dai Y. Ran N. Yin H. F. (2018). Serum exosomes can restore cellular function *in vitro* and be used for diagnosis in dysferlinopathy. Theranostics 8, 1243–1255. 10.7150/thno.22856 29507617 PMC5835933

[B9] Evans C. E. Iruela-Arispe M. L. Zhao Y. Y. (2021). Mechanisms of endothelial regeneration and vascular repair and their application to regenerative medicine. Am. J. Pathol. 191, 52–65. 10.1016/j.ajpath.2020.10.001 33069720 PMC7560161

[B10] Famta P. Shah S. Khatri D. K. Guru S. K. Singh S. B. Srivastava S. (2022). Enigmatic role of exosomes in breast cancer progression and therapy. Life Sci. 289, 120210. 10.1016/j.lfs.2021.120210 34875250

[B11] Fu Y. J. Xu B. Huang S. W. Luo X. Deng X. L. Luo S. (2020). Baicalin prevents LPS-induced activation of TLR4/NF-κB p65 pathway and inflammation in mice *via* inhibiting the expression of CD14. Acta Pharmacol. Sin. 42, 88–96. 10.1038/s41401-020-0411-9 32457419 PMC7921675

[B12] Gao M. Yu T. Y. Liu D. Shi Y. Yang P. L. Zhang J. (2021). Sepsis plasma-derived exosomal miR-1-3p induces endothelial cell dysfunction by targeting SERP1. Clin. Sci. 135, 347–365. 10.1042/CS20200573 33416075 PMC7843403

[B13] Gliozzi M. Scicchitano M. Bosco F. Musolino V. Carresi C. Scarano F. (2019). Modulation of nitric oxide synthases by oxidized LDLs: role in vascular inflammation and atherosclerosis development. Int. J. Mol. Sci. 20, 3294. 10.3390/ijms20133294 31277498 PMC6651385

[B14] Gupta R. M. Libby P. Barton M. (2020). Linking regulation of nitric oxide to Endothelin-1: the yin and Yang of vascular tone in the atherosclerotic plaque. Atherosclerosis 292, 201–203. 10.1016/j.atherosclerosis.2019.11.001 31810569 PMC7447109

[B15] Han C. S. Yang J. J. Sun J. C. Qin G. J. (2022). Extracellular vesicles in cardiovascular disease: biological functions and therapeutic implications. Pharmacol. Ther. 233, 108025. 10.1016/j.pharmthera.2021.108025 34687770 PMC9018895

[B16] Heo J. Kang H. (2022). Exosome-based treatment for atherosclerosis. Int. J. Mol. Sci. 23, 1002. 10.3390/ijms23021002 35055187 PMC8778342

[B17] Hou Z. X. Qin X. H. Hu Y. Y. Zhang X. Li G. H. Wu J. (2019). Longterm exercise-derived exosomal miR-342-5p: a novel exerkine for cardioprotection. Circ. Res. 124, 1386–1400. 10.1161/CIRCRESAHA.118.314635 30879399

[B18] Hu Q. Yao J. Q. Wu X. J. Li J. Li G. X. Tang W. F. (2022). Emodin attenuates severe acute pancreatitis-associated acute lung injury by suppressing pancreatic exosome-mediated alveolar macrophage activation. Acta. Pharm. Sin. B 12, 3986–4003. 10.1016/j.apsb.2021.10.008 36213542 PMC9532455

[B19] Isaac R. Reis F. C. G. Ying W. Olefsky J. M. (2021). Exosomes as mediators of intercellular crosstalk in metabolism. Cell Metab. 33, 1744–1762. 10.1016/j.cmet.2021.08.006 34496230 PMC8428804

[B20] Jin Y. H. Liu G. Q. Yu Q. Q. Ma S. M. Chang M. (2022). Serum extracellular vesicles attenuate cardiomyocyte injury induced by hypoxic/reoxygenation by regulating miR-1229-5p. Tohoku. J. Exp. Med. 258, 35–41. 10.1620/tjem.2022.J048 35705319

[B21] Li W. X. Wang X. Y. Jia W. H. Zhang M. L. Tang J. F. Li X. L. (2021). Effects of active components of naoxintong capsule on the JAK/STAT signal pathway, vasoactive substances, adhesion molecules and inflammatory factors of HUVEC. China Pharm. 32, 301–308. 10.6039/j.issn.1001-0408.2021.03.09

[B22] Li Y. P. Pan X. Yin M. Y. Li C. P. Han L. R. (2021). Preventive effect of lycopene in dextran sulfate sodium-induced ulcerative colitis mice through the regulation of TLR4/TRIF/NF-κB signaling pathway and tight junctions. J. Agric. Food Chem. 69, 13500–13509. 10.1021/acs.jafc.1c05128 34729976

[B23] Li S. H. Zhang Y. Y. Sun Y. L. Zhao H. J. Wang Y. (2021). Inhibition of microRNA-802-5p inhibits myocardial apoptosis after myocardial infarction *via* sonic hedgehog signaling pathway by targeting PTCH1. Eur. Rev. Med. Pharmacol. Sci. 25, 326–334. 10.26355/eurrev_202101_24398 33506921

[B24] Li R. Yang H. Xue Y. Luo X. Q. (2024). Yemazhui ameliorates lipopolysaccharide-induced acute lung injury modulation of the toll-like receptor 4/Nuclear factor Kappa-B/Nod-Like receptor family pyrin domain-containing 3 protein signaling pathway and intestinal flora in rats. J. Tradit. Chin. Med. 44, 303–314. 10.19852/j.cnki.jtcm.20230510.001 38504536 PMC10927412

[B25] Liu J. J. Jiang C. Y. Ma X. Wang J. B. (2018). Notoginsenoside Fc attenuates high glucose-induced vascular endothelial cell injury *via* upregulation of PPAR-γ in diabetic sprague-dawley rats. Vasc. Pharmacol. 109, 27–35. 10.1016/j.vph.2018.05.009 29857059

[B26] Liu Y. Sun Y. Lin X. Z. Zhang D. Hu C. P. Liu J. X. (2022). Perivascular adipose-derived exosomes reduce macrophage foam cell formation through miR-382-5p and the BMP4-PPARγ-ABCA1/ABCG1 pathways. Vasc. Pharmacol. 143, 106968. 10.1016/j.vph.2022.106968 35123060

[B27] Lu Y. H. Wan H. F. Wu Y. J. Yang J. H. Yu L. He Y. (2022). Naoxintong capsule alternates gut microbiota and prevents hyperlipidemia in high-fat-diet fed rats. Front. Pharmacol. 13, 843409. 10.3389/fphar.2022.843409 35387330 PMC8978017

[B28] Ma D. (2023). Study on myocardial protection in ischemia/reperfusion injury by intervention of Qingxin Jieyu formula with exosomes-pyroptosis. China Academy of Chinese Medical Sciences, 145. Doctoral Dissertation. 10.27658/d.cnki.gzzyy.2023.000134

[B29] Marongiu L. Gornati L. Artuso I. Zanoni I. Granucci F. (2019). Below the surface: the inner lives of TLR4 and TLR9. J. Leukoc. Biol. 106, 147–160. 10.1002/JLB.3MIR1218-483RR 30900780 PMC6597292

[B30] Mattingly A. J. Laitano O. Garcia C. K. Robinson G. P. Clanton T. L. (2022). Lipopolysaccharide-induced cytokine secretion from *in vitro* mouse slow and fast limb muscle. Shock 57, 600–607. 10.1097/SHK.0000000000001891 34798635 PMC8917056

[B31] Miao J. Q. Shen J. Yan C. Q. Ren J. H. Liu H. X. Qiao Y. B. (2022). The protective effects of mai-luo-ning injection against LPS-induced acute lung injury *via* the TLR4/NF-κB signalling pathway. Phytomedicine 104, 154290. 10.1016/j.phymed.2022.154290 35793597

[B32] Papini G. Furini G. Matteucci M. Biemmi V. Casieri V. Di Lascio N. (2023). Cardiomyocyte-targeting exosomes from sulforaphane-treated fibroblasts affords cardioprotection in infarcted rats. J. Transl. Med. 21, 313. 10.1186/s12967-023-04155-x 37161563 PMC10169450

[B33] Scicchitano P. Cameli M. Maiello M. Modesti P. A. Muiesan M. L. Novo S. (2014). Nutraceuticals and dyslipidaemia: beyond the common therapeutics. J. Funct. Foods. 6, 11–32. 10.1016/j.jacl.2023.05.099

[B34] Sekhavati N. Noori E. Abbasifard M. Butler A. E. Sahebkar A. (2022). How Statin drugs affect exosomes? J. Cell. Biochem. 124, 171–180. 10.1002/jcb.30363 36565475

[B35] Shi M. M. Yuan Y. J. Liu J. P. Chen Y. N. Li L. Liu S. Y. (2018). MSCs protect endothelial cells from inflammatory injury partially by secreting STC1. Int. Immunopharmacol. 61, 109–118. 10.1016/j.intimp.2018.05.016 29857240

[B36] Shi Z. M. Zhu Q. Fan J. M. (2021). lncRNA TUG1 promotes atherosclerosis progression by targeting miR-382-5p. Int. J. Clin. Exp. Pathol. 14, 972–979. 34646415 PMC8493262

[B37] Shu J. Huang R. Z. Tian Y. Liu Y. R. Zhu R. Shi G. (2020). Andrographolide protects against endothelial dysfunction and inflammatory response in rats with coronary heart disease by regulating PPAR and NF-κB signaling pathways. Ann. Palliat. Med. 9, 1965–1975. 10.21037/apm-20-960 32692217

[B38] Tong Y. Zhou M. H. Li S. P. Zhao H. M. Zhang Y. R. Chen D. (2023). MiR-155-5p attenuates vascular smooth muscle cell oxidative stress and migration *via* inhibiting BACH1 expression. Biomedicines 11, 1679. 10.3390/biomedicines11061679 37371773 PMC10295705

[B39] Vaidya M. Sugaya K. (2020). DNA associated with circulating exosomes as A biomarker for glioma. Genes 11, 1276. 10.3390/genes11111276 33137926 PMC7692052

[B40] Wang Y. Q. Yan X. X. Mi S. L. Li Z. Wang Y. X. Zhu H. (2016). Naoxintong Attenuates ischaemia/reperfusion injury through inhibiting NLRP3 inflammasome activation. J. Cell Mol. Med. 21, 4–12. 10.1111/jcmm.12915 27785882 PMC5192872

[B41] Wang Z. H. Liu P. R. Hu M. Y. Lu S. X. Lyu Z. J. Kou Y. (2021). Naoxintong restores ischemia injury and inhibits thrombosis *via* COX2-VEGF/NFκB signaling. J. Ethnopharmacol. 270, 113809. 10.1016/j.jep.2021.113809 33444716

[B42] Wang P. Li X. L. Cao Z. H. (2021). STC1 ameliorates cognitive impairment and neuroinflammation of Alzheimer's disease mice *via* inhibition of ERK1/2 pathway. Immunobiology 226, 152092. 10.1016/j.imbio.2021.152092 34004549

[B43] Wang Y. Xu Y. H. Zhang L. Huang S. W. Dou L. P. Yang J. H. (2022). Comparison of Buyang Huanwu granules and naoxintong capsules in the treatment of stable angina pectoris: rationale and design of A randomized, blinded, multicentre clinical trial. Trials 23, 65. 10.1186/s13063-021-05914-1 35062988 PMC8780317

[B44] Watanabe M. Horie H. Kurata Y. Inoue Y. Notsu T. Wakimizu T. (2021). Esm1 and Stc1 as angiogenic factors responsible for protective actions of adipose-derived stem cell sheets on chronic heart failure after rat myocardial infarction. Circ. J. 85, 657–666. 10.1253/circj.CJ-20-0877 33716265

[B45] Wei H. B. Qian X. Q. Xie F. Cui D. X. (2021). Isolation of exosomes from serum of patients with lung cancer: a comparison of the ultra-high speed centrifugation and precipitation methods. Ann. Transl. Med. 9, 882. 10.21037/atm-21-2075 34164516 PMC8184444

[B46] Wu Y. Su S. A. Xie Y. Shen J. Zhu W. Xiang M. X. (2018). Murine models of vascular endothelial injury: techniques and pathophysiology. Thromb. Res. 169, 64–72. 10.1016/j.thromres.2018.07.014 30015230

[B47] Xiao Y. C. Wang W. Gao Y. Li W. Y. Tan X. Wang Y. K. (2022). The peripheral circulating exosomal MicroRNAs related to central inflammation in chronic heart failure. J. Cardiovasc. Transl. Res. 15, 500–513. 10.1007/s12265-022-10266-5 35501543

[B48] Xu F. Yao F. Ning Y. Y. (2023). MicroRNA-202-5p-Dependent inhibition of Bcl-2 contributes to macrophage apoptosis and atherosclerotic plaque formation. Gene 867, 147366. 10.1016/j.gene.2023.147366 36931409

[B49] Xue P. Huang S. H. Han X. Zhang C. Y. Yang L. Xiao W. F. (2022). Exosomal miR-101-3p and miR-423-5p inhibit medulloblastoma tumorigenesis through targeting FOXP4 and EZH2. Cell Death Differ. 29, 82–95. 10.1038/s41418-021-00838-4 34294888 PMC8738741

[B50] Yan Z. H. Zhang W. J. Wu H. Chen T. B. He Y. Su W. W. (2021). Progress in the material basis, and effects and mechanisms of naoxintong capsule on metabolic disturbance-related diseases. Acta Sci. Nat. Univ. Sunyatseni 60, 12–18. 10.13471/j.cnki.acta.snus.2020.08.05.2020e033

[B51] You L. J. Zhang D. Geng H. Sun F. Y. Lei M. (2021). Salidroside protects endothelial cells against LPS-induced inflammatory injury by inhibiting NLRP3 and enhancing autophagy. BMC Complement. Med. Ther. 21, 146. 10.1186/s12906-021-03307-0 34011327 PMC8136193

[B52] Zhang H. Liu J. Qu D. Wang L. Wong C. M. Lau C. W. (2018). Serum exosomes mediate delivery of arginase 1 as A novel mechanism for endothelial dysfunction in diabetes. Proc. Natl. Acad. Sci. USA. 115, E6927–E6936. 10.1073/pnas.1721521115 29967177 PMC6055191

[B53] Zhang W. J. Su W. W. Li P. B. Rao H. Y. Lin Q. W. Zeng X. (2019). Naoxintong capsule inhibits the development of cardiovascular pathological changes in bama minipig through improving gut microbiota. Front. Pharmacol. 10, 1128. 10.3389/fphar.2019.01128 31632272 PMC6785636

[B54] Zhang W. J. Su W. W. Lin Q. W. He Y. Yan Z. H. Wang Y. G. (2020). Protective effects of naoxintong capsule alone and in combination with ticagrelor and atorvastatin in rats with Qi deficiency and blood stasis syndrome. Pharm. Biol. 58, 1006–1022. 10.1080/13880209.2020.1821066 32985308 PMC7534269

[B55] Zhang F. M. Zheng W. H. Wang H. J. (2020). MiR-34a-5p inhibition attenuates LPS-induced endothelial cell injury by targeting FOXM1. Eur. Rev. Med. Pharmacol. Sci. 24, 10829–10838. 10.26355/eurrev_202010_23445 33155244

[B56] Zhang L. Q. Zhu H. J. Teng X. L. Sheng X. S. Yu B. W. (2021). Modulation of miR-382-5p reduces apoptosis of myocardial cells after acute myocardial infarction. Autoimmunity 54, 1–9. 10.1080/08916934.2021.1910812 34042547

[B57] Zhang W. J. Chen R. Q. Tang X. Li P. B. Wang J. Wu H. K. (2024). Naoxintong capsule for treating cardiovascular and cerebrovascular diseases: from bench to bedside. Front. Pharmacol. 15, 1402763. 10.3389/fphar.2024.1402763 38994201 PMC11236728

[B58] Zhou Y. Q. Zhao Y. T. Zhao X. Y. Liang C. Xu Y. W. Li L. (2018). Hyperoside suppresses lipopolysaccharide-induced inflammation and apoptosis in human umbilical vein endothelial cells. Curr. Med. Sci. 38, 222–228. 10.1007/s11596-018-1869-2 30074179

