# Correction: Naoxintong capsule decreases circulating exosomes of miR-382-5p to protect LPS-induced vascular endothelial cell injury by targeting STC1 *in vitro*

**DOI:** 10.3389/fphar.2026.1827794

**Published:** 2026-04-21

**Authors:** Ziyan Xu, Hao Wu, Weiyang Fan, Fenzhao Meng, Shuangfei Hu, Muhan Zou, Yixuan Chen, Weiwei Su, Peibo Li

**Affiliations:** Guangdong Engineering and Technology Research Center for Quality and Efficacy Re-Evaluation of Post-Marketed TCM, Guangdong Provincial Key Laboratory of Plant Stress Biology, State Key Laboratory of Biocontrol, School of Life Sciences, Sun Yat-sen University, Guangzhou, China

**Keywords:** circulating exosomes, miR-382-5p, naoxintong capsule (NXT), protective effect, stanniocalcin-1 (STC1), TLR4/TRIF/NF-κB pathway, vascular endothelial injury

In the original publication, there were a few mistakes in Figures 1, 2, 7, 8. In Figure 1D, the kDa value for the protein ALIX was incorrectly stated as “80” instead of the correct “96”. In Figure 2G, the kDa value for the protein Cleaved caspase 3 was incorrectly stated as “23” instead of the correct “17”. In Figure 7I, the kDa value for the protein Cleaved caspase 3 was incorrectly stated as “16” instead of the correct “17”. In Figure 8H, the kDa value for the protein Cleaved caspase 3 was incorrectly stated as “23” instead of the correct “17”. The corrected Figures 1, 2, 7, 8 appear below.

**FIGURE 1:**
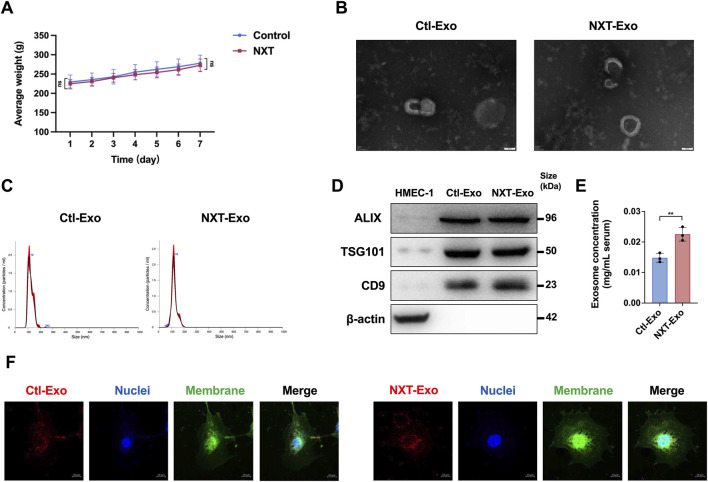
Body weight map and biological identification, concentration determination, and internalization of circulating exosomes. **(A)** Daily body weight of SD rats during the experiment. **(B)** Circulating exosomes scanning results of TEM (Scale bar, 50 nm). **(C)** The particle diameter distribution of circulating exosomes tested by NTA. **(D)** The circulating exosome markers (ALIX, TSG101, CD9) and the negative control marker (β-actin) detected by Western blot. **(E)** The concentrations of circulating exosomes tested by BCA. **(F)** The internalization of circulating exosomes from SD rats in HMEC-1 imaged by confocal microscopy (Scale bar, 10 μm). PKH-26 (Ex: 551 nm, Em: 562∼580 nm) labeled circulating exosomes, Dio (Ex: 484 nm, Em: 496∼520 nm) labeled cell membrane, and Hoechst 33342 (Ex: 346 nm, Em: 455∼470 nm) labeled cell nuclei. Values are expressed as mean ± SD, n = 3. ^*^ *p* < 0.05, ^**^ *p* < 0.01 and ns indicated no significant statistical difference.

**FIGURE 2:**
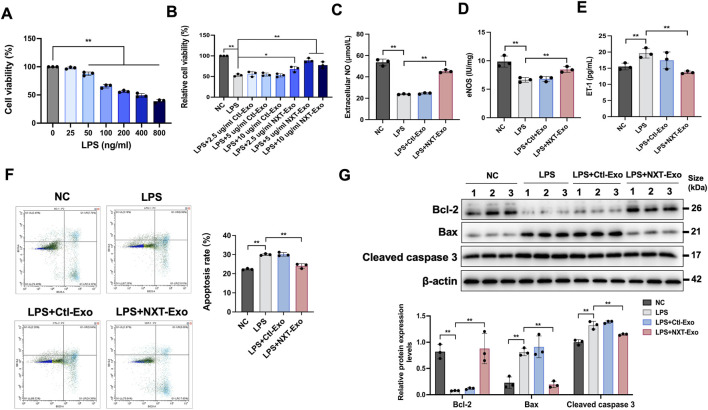
NXT-Exo inhibited LPS-induced HMEC-1 dysfunction and apoptosis. **(A)** The cell viability of HMEC-1 after LPS stimulation were detected by CCK-8 assay. **(B)** The effects of NXT-Exo on the cell activity of HMEC-1 induced by LPS were detected by CCK-8 assay. **(C–E)** The expression levels of NO, eNOS and ET-1 in HMEC-1 were measured by ELISA. **(F)** The apoptosis rates of HMEC-1 were detected by flow cytometry. The horizontal axis B525-A corresponds to the detection of Annexin V staining (Ex = 494 nm, Em = 518 nm), and the vertical axis B610-A corresponds to the detection of PI staining (Ex = 535 nm, Em = 617 nm). **(G)** The relative protein expression levels of Bax, Bcl-2 and Cleaved caspase 3 were detected by Western blot. Values are expressed as mean ± SD, n = 3. ^*^ *p* < 0.05 and ^**^ *p* < 0.01.

**FIGURE 7:**
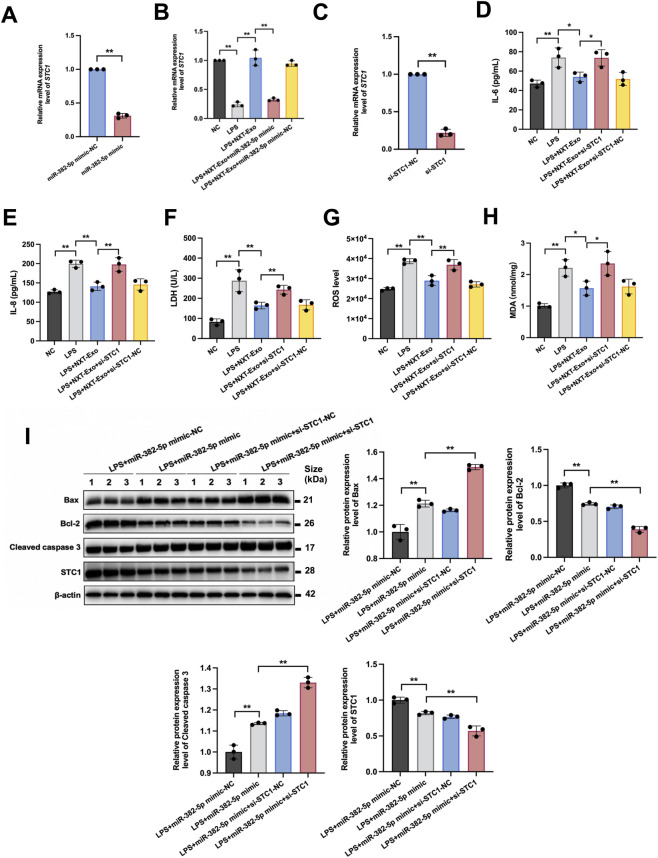
miR-382-5p downregulated the expression of STC1 and the effect of STC1 on the endothelial protection of NXT-Exo. **(A)** The mRNA expression level of STC1 in HMEC-1 transfected with miR-382-5p mimic or miR-382-5p mimic-NC was detected by RT-qPCR. **(B)** The mRNA expression level of STC1 in HMEC-1 treated with NXT-Exo or NXT-Exo combined with miR-382-5p was detected by RT-qPCR. **(C)** The transfection of si-STC1 was verified by RT-qPCR. **(D,E)** The secretion levels of IL-6 and IL-8 were measured by ELISA. **(F)** The release of LDH was detected by microplate method. **(G,H)** The production level of ROS and expression level of MDA were tested by fluorescence. **(I)** The effects of miR-382-5p and STC1 treatment on the relative protein expression levels of Bax, Bcl-2, Cleaved caspase 3 and STC1 in HMEC-1 were detected by Western blot. Values are expressed as mean ± SD, n = 3. ^*^ *p* < 0.05, ^**^ *p* < 0.01.

**FIGURE 8:**
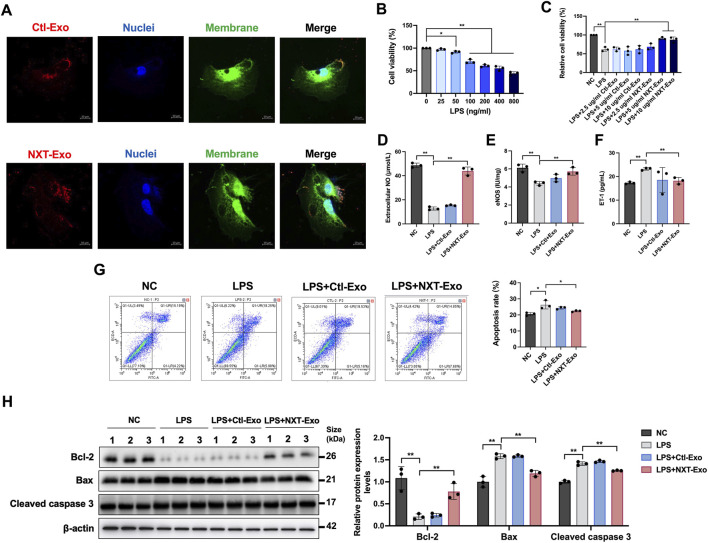
NXT-Exo inhibited LPS-induced HUVEC dysfunction and apoptosis. **(A)** The internalization of circulating exosomes from SD rats in HUVEC imaged by confocal microscopy (Scale bar, 10 μm). PKH-26 (Ex: 551 nm, Em: 562∼580 nm) labeled circulating exosomes, Dio (Ex: 484 nm, Em: 496∼520 nm) labeled cell membrane, and Hoechst 33342 (Ex: 346 nm, Em: 455∼470 nm) labeled cell nuclei. **(B)** The cell viability of HUVEC after LPS stimulation were detected by CCK-8 assay. **(C)** The effects of NXT-Exo on the cell activity of HUVEC induced by LPS were detected by CCK-8 assay. **(D–F)** The expression levels of NO, eNOS and ET-1 in HUVEC were measured by ELISA. **(G)** The apoptosis rates of HUVEC were detected by flow cytometry. The horizontal axis B525-A corresponds to the detection of Annexin V staining (Ex = 494 nm, Em = 518 nm), and the vertical axis B610-A corresponds to the detection of PI staining (Ex = 535 nm, Em = 617 nm). **(H)** The relative protein expression levels of Bax, Bcl-2 and Cleaved caspase 3 were detected by Western blot. Values are expressed as mean ± SD, n = 3. ^*^ *p* < 0.05, ^**^ *p* < 0.01.

The original article has been updated.

